# Integrated analysis of cytochrome P450 gene superfamily in the red flour beetle, *Tribolium castaneum*

**DOI:** 10.1186/1471-2164-14-174

**Published:** 2013-03-14

**Authors:** Fang Zhu, Timothy W Moural, Kapil Shah, Subba Reddy Palli

**Affiliations:** 1 Department of Entomology, College of Agriculture, University of Kentucky, Lexington, KY, 40546, USA

**Keywords:** Insecticide resistance, Gene cluster, Molecular docking, Mitochondrial CYPs

## Abstract

**Background:**

The functional and evolutionary diversification of insect cytochrome P450s (CYPs) shaped the success of insects. CYPs constitute one of the largest and oldest gene superfamilies that are found in virtually all aerobic organisms. Because of the availability of whole genome sequence and well functioning RNA interference (RNAi), the red flour beetle, *Tribolium castaneum* serves as an ideal insect model for conducting functional genomics studies. Although several *T. castaneum* CYPs had been functionally investigated in our previous studies, the roles of the majority of CYPs remain largely unknown. Here, we comprehensively analyzed the phylogenetic relationship of all *T. castaneum* CYPs with genes in other insect species, investigated the *CYP6BQ* gene cluster organization, function and evolution, as well as examined the mitochondrial CYPs gene expression patterns and intron-exon organization.

**Results:**

A total 143 CYPs were identified and classified into 26 families and 59 subfamilies. The phylogenetic trees of CYPs among insects across taxa provided evolutionary insight for the genetic distance and function. The percentage of singleton (33.3%) in *T. castaneum* CYPs is much less than those in *Drosophila melanogaster* (52.5%) and *Bombyx mori* (51.2%). Most members in the largest CYP6BQ gene cluster may make contribution to deltamethrin resistance in QTC279 strain. *T. castaneum* genome encodes nine mitochondrial CYPs, among them *CYP12H1* is only expressed in the final instar larval stage. The intron-exon organizations of these mitochondrial CYPs are highly diverse.

**Conclusion:**

Our studies provide a platform to understand the evolution and functions of *T. castaneum* CYP gene superfamily which will help reveal the strategies employed by insects to cope with their environment.

## Background

Insects appeared more than 450 million years ago [1,2] and have been known to be the unprecedented evolutionally successful metazoans on the earth. One of the factors that may contribute to this success is the ability of insects to adapt to almost every ecological niche by virtue of traits such as metamorphosis and flight [3,4]. In the meantime, the radiation of insects into diverse habitats and food sources largely enhanced the risk for them to be exposed to toxic or otherwise life-threatening conditions. Insect CYPs impact on the ability of insect adaptation to diverse habitats. On one hand, CYPs have very important physiological functions during all life stages of insects. They might be involved in the biosynthesis pathway of endogenous compounds, such as molting hormone (20-hydroxyecdysone, 20E is the most active form) [3] and juvenile hormone (JH) [5] that are the key factors in regulating metamorphosis, development, and reproduction. Some insect CYPs are also involved in the degradation of pheromones [6,7] as well as catalysis and hydroxylation of fatty acids [8], which are critical for chemical communication, behavior [7,9] and metabolism. On the other hand, as a group of environmental response genes [10], some CYPs protect insects by detoxifying xenobiotics including synthetic insecticides [11-13] and plant allelochemicals [14,15], resulting in the adaption of insects to the chemical stresses. In a way, the functional and evolutionary diversification (“bloom”) of insect CYPs has shaped the success of insects.

As microsomal pigments, CYPs have an absorption peak at 450 nm when reduced and saturated with carbon monoxide [16]. The bacterial CYPs are water soluble while eukaryotic CYPs are membrane-bound proteins located either on the endoplasmic reticulum or the inner mitochondrial membrane. P450s are hemoproteins and act as the terminal oxidases in the monooxygenase system [12]. The three components of the P450 monooxygenase system are P450, which acts as the substrate binding protein, NADPH-cytochrome P450 reductase (CPR), which transfers electrons from NADPH to CYPs, and cytochrome b5, which transfers electrons from NADH to CYPs in some P450 monooxygenase systems as an additional potential electron donor [11].

CYPs constitute one of the largest and oldest gene superfamilies that are found in virtually all aerobic organisms [17]. Insects typically contain tens to more than one hundred individual P450 genes in their genomes (http://drnelson.uthsc.edu/CytochromeP450.html). Genome annotation efforts identified 90 CYPs in *D. melanogaster*[18], 111 CYPs in *Anopheles gambiae*[19], 84 CYPs in *B. mori*[20], 48 CYPs in *Apis mellifera*[21], 164 CYPs in *Aedes aegypti*[22], 204 CYPs in *Culex quinquefasciatus*[23], 38 CYPs in *Pediculus humanus humanus*[24], and 143 CYPs in *T. castaneum*[25]. *T. castaneum*, commonly known as the red flour beetle, is the first beetle having its genome sequenced. *T. castaneum* is a notorious worldwide pest of stored grains and farinaceous materials [26,27]. It has developed resistance to all five classes of insecticides and fumigants used against it [25]. Moreover, the functional genomics method, RNAi, works systemically in almost every tissue and developmental stage of *T. castaneum*[28,29]. These characters make *T. castaneum* an ideal insect model for conducting functional genomics, investigating the mechanisms of insecticide resistance, and exploiting potential new insecticide targets for pest control. Although several *T. castaneum* CYPs, *CYP6BQ9*[30], *CYP306A1*[31-33], *CYP314A1*[31-33] had been functionally investigated in our previous studies, the role of the majority of CYPs remains largely unknown. Here, we analyzed the phylogenetic relationship of all *T. castaneum* CYPs with genes in other insect species, examined the CYP6BQ gene cluster organization, function and evolution, as well as investigated the mitochondrial CYPs intron-exon and gene expression patterns which provide platform to understand the evolution and predict the functions of *T. castaneum* P450 genes.

## Results and discussion

### Annotation of *T. castaneum* CYPs

Total 143 *T. castaneum* CYPs were annotated and analyzed based on the NCBI database (http://www.ncbi.nlm.nih.gov), Beetlebase (http://beetlebase.org/) and Cytochrome P450 homepage (http://drnelson.uthsc.edu/CytochromeP450.html). A full *T. castaneum* P450 gene list with the most updated assembly and annotation information including clan, name, accession number, symbol synonyms, map position and amino acid length are provided in the Additional file 1[34]. Among these 143 genes, 133 genes are putatively functional isoforms, and 10 are pseudogenes. These genes fall into four clans, clan 2, mitochondrial clan, clan 3, and clan 4 (see Additional file 1, Table 1). These four clans are further classified into 26 families and 59 subfamilies. Nine new families were discovered including mitochondrial family CYP353, CYP3 clan families CYP345, 346, 347, and 348, and CYP4 clan families CYP349, 350, 351, and 352 (see Additional file 1, Table 1).

**Table 1:** **Number of *T. castaneum *CYP families, subfamilies, pseudogenes, and genes in each insect P450 clan**

**Number**	**CYP2**	**Mitochondrial**	**CYP3**	**CYP4**	**Total**
Family	7 (CYP15, 18, 303-307)	8 (CYP12, 49, 301, 302, 314, 315, 334, 353)	6 (CYP6, 9, 345-348)	5 (CYP4, 349-352)	26
Subfamily	8	9	27	15	59
Pseudogenes	0	0	7	3	10
All genes	8	9	79	47	143

### Phylogenetic analysis of *T. castaneum* CYPs

**Figure 1:**
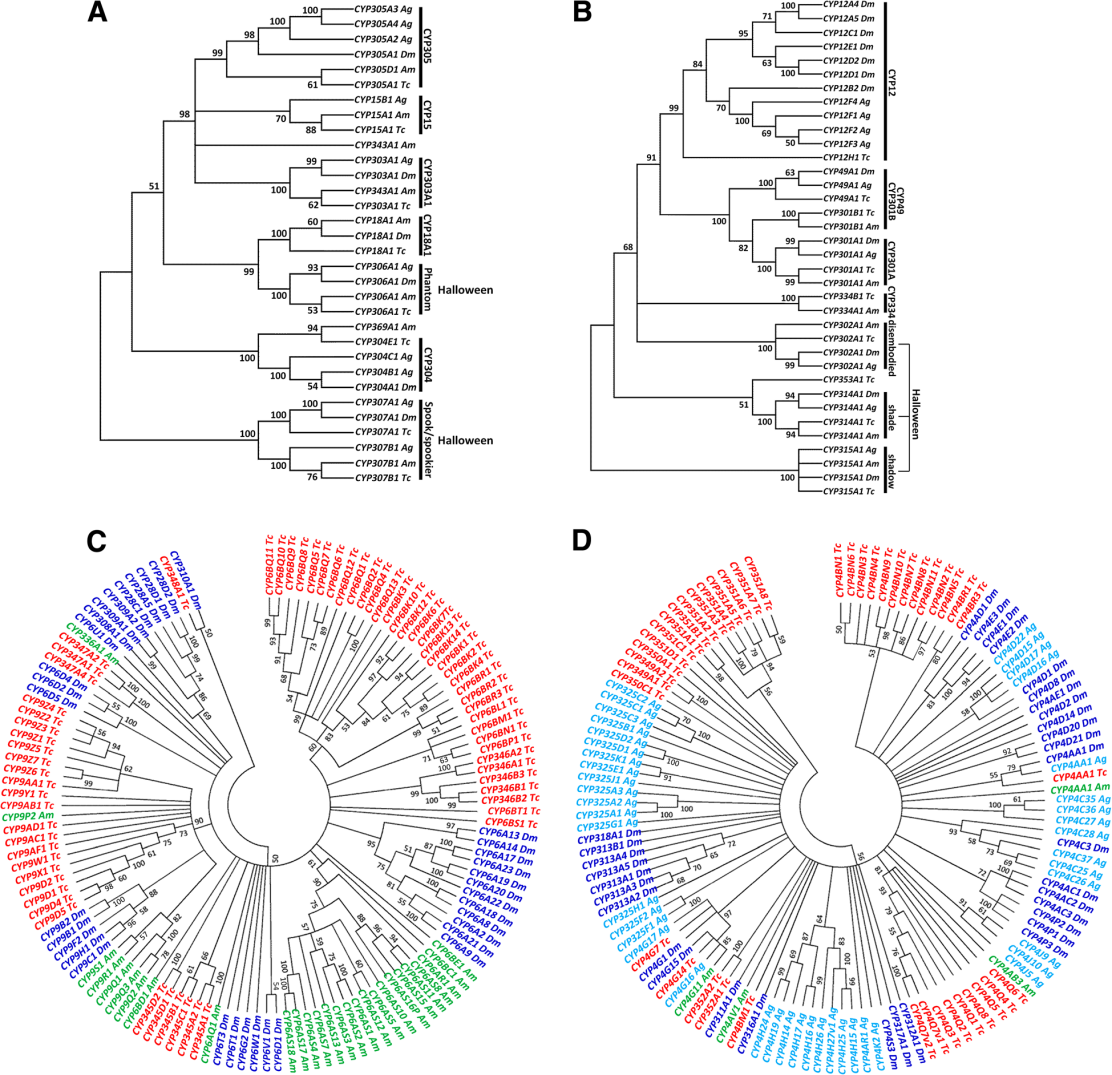
**Neighbor-joining consensus trees of the four P450 clans.** (**A**) CYP2; (**B**) mitochondrial; (**C**) CYP3; and (**D**) CYP4. The phylogenetic trees were generated by MEGA 5 using the amino acid sequences from *T. castaneum* (Tc), ***D****. melanogaster* (Dm), *A. gambiae* (Ag), and *A. mellifera* (Am). Two-letter species designations followed the specific gene names. All nodes have significant bootstrap support based on 2,000 replicates. The trees were created with cut-off value of 50%.

To inspect the evolutionary relationships of CYPs among insect CYPomes across taxa which might provide evolutionary insight for the genetic distance and function, four phylogenetic trees were constructed with CYPs identified in *T. castaneum*, *D. melanogaster*, *A. gambiae*, and *A. mellifera* (Figures 1A, B, C, D). *T. castaneum* P450s CYP2 and mitochondrial clans present a high level of 1:1 orthology with those from other insect genomes, suggesting functional conservation of these CYPs [1]. Within CYP2 clan, two out of eight genes (*CYP303A1* and *CYP306A1*) show precise 1:1:1:1 orthologies (Figure 1A). In *D. melanogaster*, CYP303A1 encoded by the gene *nompH* is expressed specifically in the sensory bristles; this gene product is known to play essential roles in the development of external sensory organs associated with the reception of vital mechanosensory and chemosensory stimuli [35]. CYP306A1 encoded by *Phantom* (or *Phm*) and expressed in the prothoracic glands of *D. melanogaster* and *B. mori* was demonstrated to be involved in the ecdysteroid biosynthesis [36,37]. In *T. castaneum*, the *CYP306A1* mRNA levels showed a similar pattern as the ecdysteroids titer during five days after adult emergence in male beetles, indicating a possible function in the ecdysteroid biosynthesis [32]. However, the *CYP306A1* RNAi did not block the primary oocyte maturation which is regulated by ecdysteroids in female beetles [33]. There are three other clades in CYP2 clan for which functions had been investigated. *CYP18A1* with 26-hydroxylase activity in *D. melanogaster* is essential for proper insect development [38]. The *Spook/Spookier* CYPs are involved in ecdysteroid biosynthesis [39,40] and CYP15A1 was characterized as an ortholog of the juvenile hormone epoxidase in the cockroach [5].

Within mitochondrial clan, four out of nine *T. castaneum* CYPs show distinct 1:1:1:1 orthologies with genes from other insect species (Figure 1B). The three CYPs CYP302A1, CYP314A1, and CYP315A1 encoded by *D. melanogaster* Halloween genes *disembodied* (*dib*), *shade* (*shd*), and *shadow* (*sad*) respectively are involved in ecdystroid biosynthesis [3]. Since the *T. castaneum* CYP302A1, CYP314A1, and CYP315A1 share high sequence similarity with those of *D. melanogaster*, they very likely have similar functions in *T. castaneum*. The *T. castaneum* genome contains a single *CYP12H1* gene in CYP12 family that includes genes associated with insecticide resistance in the house fly *Musca domestica*[41] and *D. melanogaster*[42].

*T. castaneum* genome encodes largely expanded CYP3 (27 subfamilies, 79 individual genes) and CYP4 (15 subfamilies, 47 individual genes) clans, especially the families 4 (27 genes), 6 (40 genes), and 9 (23 genes) (Figure 1C and D, see Additional file 1). Genes in these two clans appear to undergo exceedingly species-specific radiations. The CYP6 family is evolutionary related to vertebrate CYP3 and CYP5 families [1,43]. *T. castaneum* CYP6 family merely has one CYP6B subfamily. All CYP6 genes in *T. castaneum* and *A. gambiae* are clustered in one clade within species, whereas CYP6 genes in *D. melanogaster* clustered into several clades in the phylogenetic tree (Figure 1C). In dipteran and lepidopteran insects, a number of CYP6 genes were shown to be involved in resistance to a wide range of insecticides and detoxification of plant allochemicals through either constitutive overexpression and/or inducible expression in resistant strains [11,15,44,45]. In *T. casta-neum* deltamethrin-resistant QTC279 stain, *CYP6BQ9,* a brain-specific P450, is constitutively overexpressed in resistant strain and is responsible for the majority of delta-methrin resistance [30]. *D. melanogaster* CYP6 gene, *Cyp6a20*, is expressed in the non-neuronal support cells of olfactory sensilla associated with pheromone-sensing, and its expression level is correlated with the influence of social experience on aggressiveness [7,9].

*T. castaneum* CYP9 family is the second biggest family in the Clan 3 (Figure 1C). Several members in this family are known to be associated with insecticide resistance and metabolism of odorant compounds [46-50]. Genes in CYP4 clan show high diversity in their sequences and functions. In Clan 4, CYP4 family is the largest gene family that has members from the vertebrates and insects as well as *Caenorhabditis elegans*[13]. It is the only family in Clan 4 that has been studied in other insect species (Figure 1D, see Additional file 1). Except *CYP4AA1*, *CYP4G7*, *CYP4G14* have 1:1:1:1 orthologs in three other species, all other CYP4 genes in *T. castaneum* are clustered in several clades within species (Figure 1D). Members of family CYP4 in other insects are known to be associated with biosynthesis of endogenous compounds [51,52], pheromone metabolism [46,53], and pyrethroid insecticide resistance [49,54-56]. It is interesting that *Antheraea yamamai CYP4G25* is associated with diapauses in the pharate first instar larvae [57], indicating that the large complement of CYP4 CYPs might have much more diverse functions beyond what we appreciated, perhaps even more diversified than the CYP3 clan [13].

### Genomic distribution of *T. castaneum* CYPs

**Figure 2:**
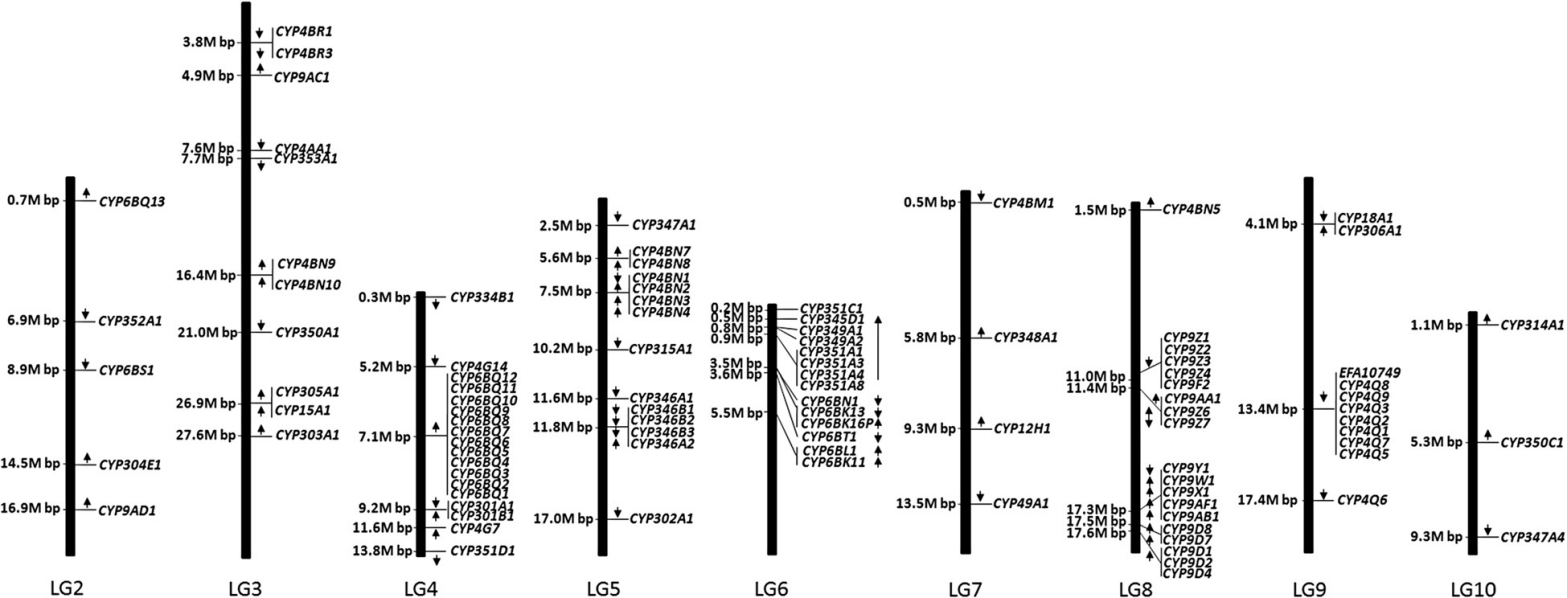
**Genetic map of the *T. castaneum *CYPs.** The physical location of each P450 gene or cluster on the chromosome map is marked on the left of the column which stands for the chromosome. Arrows indicate gene orientation: the up arrow is the reverse strand and the down arrow is the forward strand.

**Figure 3:**
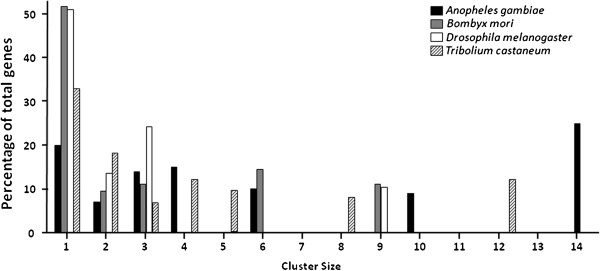
**Clusters and singletons of CYPs in the *A. gambiae*, *B. mori*, *D. melanogaster *and *T. castaneum *genomes.**

To gain a genome-wide view of chromosome location of *T. castaneum* CYPs, a genetic map (Figure 2) was constructed to map the distribution of 99 *T. castaneum* CYPs on 9 chromosomes. No P450 gene was found in the LG1=X chromosome. Majority of CYPs (87 from 99) are distributed on six chromosomes LG3, LG4, LG5, LG6, LG8 and LG9. Locations of 44 other CYPs on the chromosome remain unknown. It is considered that the formation of the substantial number of CYPs genes is due to a series of gene duplication descended from a common ancestral P450 gene [13,58-60]. Therefore, it is not surprising that most of *T. castaneum* CYPs are located on chromosomes in a tandem manner (Figures 2 and 3). There are nine clusters, defined as groups containing at least three genes, located on chromosomes LG3-6, LG8 and LG9. All the genes in these clusters belong to CYP3 and CYP4 clans; CYP6 family (LG4), CYP4 family (LG5 and LG9), CYP346 family (LG5), CYP351 family (LG6), and CYP9 family (LG8). They are remarkable landmarks for the “bloom” of the CYPome in CYP3 and CYP4 clans [2]. Only 33 out of 99 *T. castaneum* CYPs (33.3%) present as singletons. The percentage of singleton in *T. castaneum* CYPome is much less (33%) compared with CYPomes of *D. melanogaster* (52.5%) and *B. mori* (51.2%) (Figure 3) [13,20,43].

### CYP6BQ cluster in *T. castaneum*

**Figure 4:**
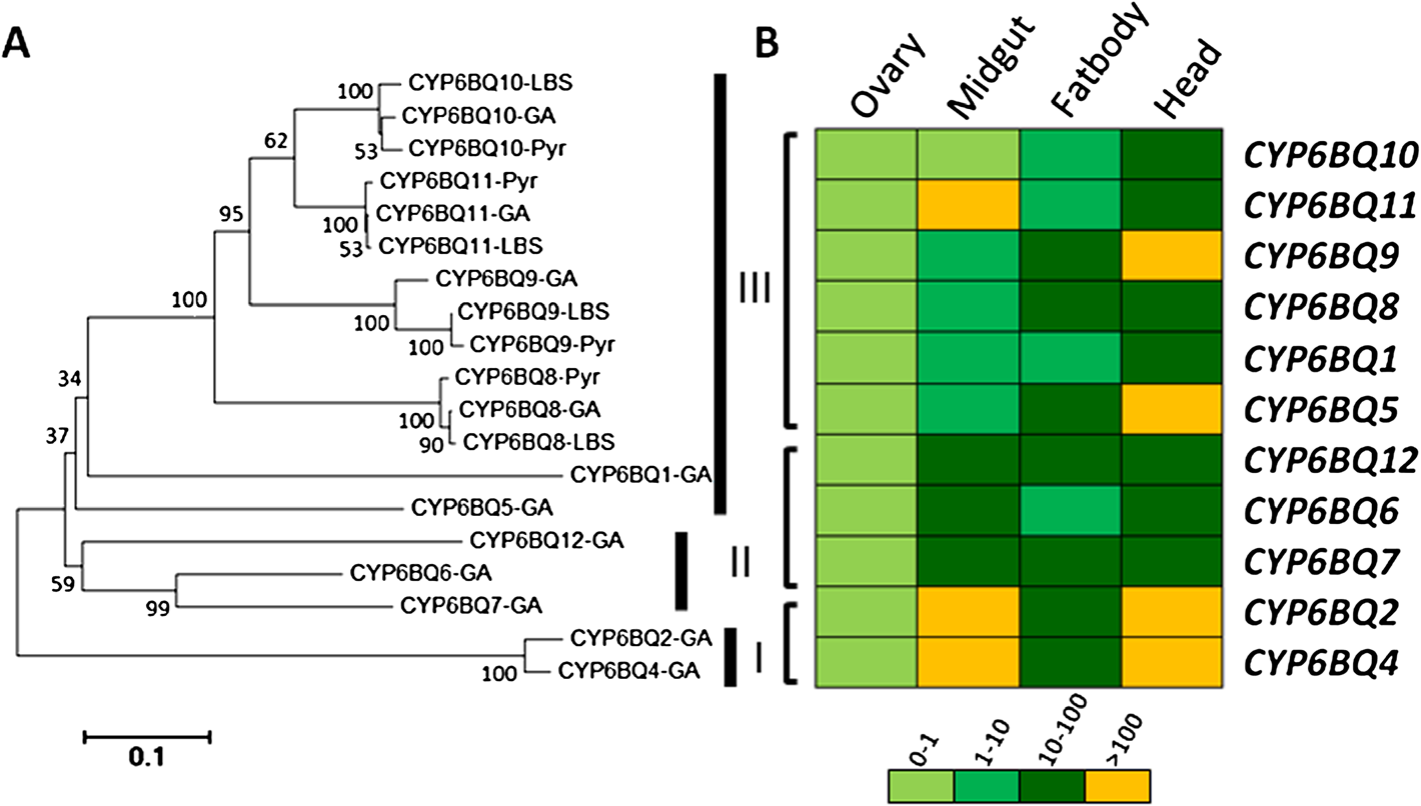
**Members in the CYP6BQ cluster.** (**A**) Neighbor-joining tree of the cluster *CYP6BQ* genes. The sequences of *CYP6BQ8*, *CYP6BQ9*, *CYP6BQ10*, and *CYP6BQ11* were cloned from QTC279 strain (Pyr), GA strain and LA strain as described in the Methods. (**B**) Tissue specific expression profile of the cluster *CYP6BQ* genes in the deltamethrinresistant QTC279 strain. The rectangles are colored on the basis of results of relative expression levels compared with that of ovary and normalized by the expression of *rp49*. The data shown are mean + SEM (n = 3). The levels of relative expression are illustrated by a four- category color scale standing for 0-1, 1-10, 10-100, >100 fold, respectively.

Clustering is a common phenomenon in the organization of CYPs in insects [11]. Around 67% of *T. castaneum* P450 genes reside in clusters (Figure 3). The largest cluster containing 12 *CYP6BQ* genes with the same orientation is located within a 30 kb region on the LG4 chromosome. One of the 12 members in this cluster, *CYP6BQ9*, had been demonstrated to be responsible for the majority of deltamethrin resistance observed in QTC279, a deltamethrin-resistant *T. castaneum* strain [30]. Further studies on the function of other members in the deltamethrin resistance might mirror the origin and evolution of this cluster. The identity matrix of genes in the CYP6BQ cluster showed amino acid homology between two cluster members ranging from 52% to 87%, with the exception of *CYP6BQ2* and *CYP6BQ4* sharing 95% amino acid identity (see Additional file 2). Figure 4A shows the phylogenetic relationships of 11 clustered genes (except the pseudogene *CYP6BQ3P*). Earlier duplication events generated three clans, which we have named clan I, II, and III. Clan I includes two genes, *CYP6BQ2* and *CYP6BQ4* with high percent sequence identities suggesting that they may have evolved from a recent duplication event. Clan II includes three genes, *CYP6BQ6*, *CYP6BQ7* and *CYP6BQ12*. Clan III consists of 6 cluster genes, *CYP6BQ1*, *CYP6BQ5*, *CYP6BQ8*, *CYP6BQ9*, *CYP6BQ10*, and *CYP6BQ11*, created by a series of tandem duplications.

The tissue specific expression pattern of genes within the same Clan is largely conserved in QTC279 strain (Figure 4B). The only exception is *CYP6BQ11*; this may imply potentially novel physiological functions for this gene. Genomic sequence analysis showed that the 12 members of *CYP6BQ* cluster share highly similar intron-exon structures (see Additional file 3). Each gene consists of three exons, with exon one having 1123-1147 bp, exon two containing about 253-256 bp, and exon three consisting of approximately 163-169 bp. There are only two exceptions, *CYP6BQ3P* containing a single exon and *CYP6BQ5* containing two exons. The length of intron ranges from about 44 bp to 54 bp. The first introns in all genes follow the canonical GT/AG rule. The differential expression profiles of the cluster genes showed that with the exception of *CYP6BQ3P* and *CYP6BQ5*, all other 10 genes have significantly higher mRNA levels in QTC279 resistant strain than in the Lab-S susceptible strain (see Additional file 4). Moreover, six out of these 10 genes were induced by deltamethrin (115 mg/48 cm^2^ that caused 50-60% mortality of beetles in QTC279 strain) after 12 h exposure to the chemical (see Additional file 5) suggesting a potential contribution of these cluster members to the deltamethrin resistance in QTC279 strain.

**Figure 5:**
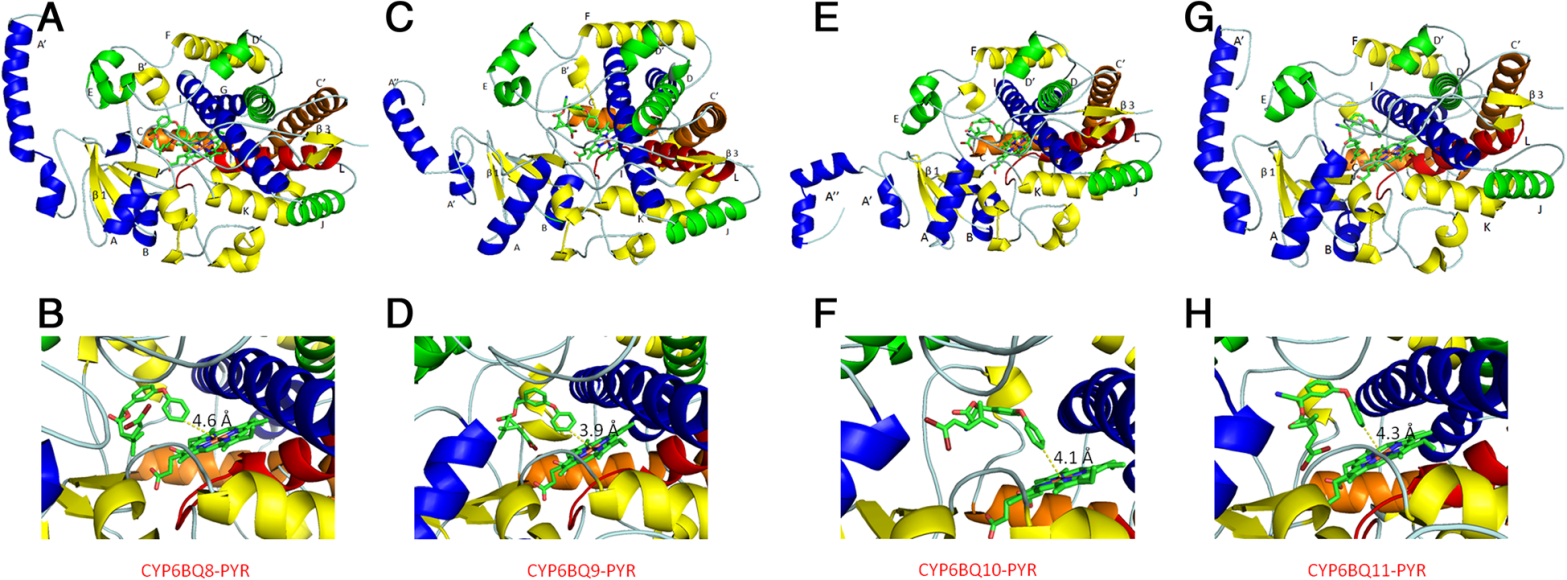
**Docking of deltamethrin in CYP6BQ catalytic sites.** Docking models for deltamethrin (elemental colors in stick format) in the predicated catalytic site of CYP6BQ8 (**A**), CYP6BQ9 (**C**), CYP6BQ10 (**E**) and CYP6BQ11 (**G**) are shown. Structurally conserved *α* helices and *β* sheets are labeled and colored to match in four models. The distances from the heme center to putative hydroxylation site of deltamethrin are shown in pictures for CYP6BQ8 (**B**), CYP6BQ9 (**D**), CYP6BQ10 (**F**), and CYP6BQ11 (**H**). Images were generated with PyMol pymol.org.

Then we focused on four evolutionarily close genes, *CYP6BQ8*, *CYP6BQ9*, *CYP6BQ10* and *CYP6BQ11*. We cloned the full length sequences of these four genes from LBS, GA and QTC279 strains and deposited them in the GenBank (CYP6BQ8-Pyr, KC686848; CYP6BQ8-GA, KC686849; CYP6BQ8-LBS, KC686850; CYP6BQ9-GA, KC686851; CYP6BQ9-LBS, KC686852; CYP6BQ10-Pyr , KC686853; CYP6BQ10-GA, KC686854; CYP6BQ10-LBS, KC686855; CYP6BQ11-Pyr, KC686856; CYP6BQ11-GA, KC686857; CYP6BQ11-LBS, KC686858). Then we conducted homology modeling and ligand docking studies. The protein models for CYP6BQ8, CYP6BQ9, CYP6BQ10 and CYP6BQ11 showed the P450 structurally conserved helices D, E, I, L, J, and K along with *β* sheets 1 and 3 (Figures 5A, C, E and G; see Additional file 6). We used AutoDock Vina to investigate the binding of four insecticide compounds DDT, imidacloprid, permethrin, and deltamethrin (see Additional file 7, Figure 5). For each predicted binding mode, estimated binding affinity and insecticide putative hydroxylation site distance from the heme iron were compared. The lowest binding energy observed was for CYP6BQ9-Pyr and deltamethrin with the 4^′^ carbon hydroxylation site adjacent to the heme iron (see Additional file 7, Figures 5C and D). All models demonstrated favorable binding affinity and a putative substrate hydroxylation site within 6.0 Å [61] of the heme iron with two or more of the insecticides tested (see Additional file 7). Relevant binding modes for CYP6BQ11-Pyr were found for all four insecticides suggesting an association with resistance to all four insecticides (see Additional file 7). Docking modes for deltamethrin are shown in Figure 5. Residues in the catalytic pockets of CYP6BQ8, CYP6BQ9, CYP6BQ10 and CYP6BQ11 within 4.5 Å of deltamethrin were examined. Hydrophobic residues Phe128, Phe248, Val320 or Leu320 and hydrophilic Lys390 were conserved in the catalytic site of at least three proteins. The active sites of all four models are rich in phenylalanine and other hydrophobic residues (see Additional file 6) suggesting these four proteins provide a favorable chemical environment for hydrophobic insecticide compounds. Although the lowest binding energy was observed for CYP6BQ9-Pyr and deltamethrin, which is consistent with our previous study about the major function of *CYP6BQ9* in deltamethrin resistance of QTC279 beetles [30], the predicted catalytic sites are well conserved among CYP6BQ9 variants in LBS, GA and QTC279 strains (see Additional file 6). The predicted binding affinities to deltamethrin and insecticide putative hydroxylation site distance from the heme iron do not differ much among CYP6BQ9 variants in these three strains (see Additional file 7). These observations further suggest that the involvement of CYP6BQ9 in deltamethrin resistance of QTC279 strain is not due to changes in binding affinity but is likely due to an increase in the expression of this gene. The mechanism of regulation of *CYP6BQ9* expression in the QTC279 strain is currently under investigation.

### Mitochondrial CYPs in *T. castaneum*

The mitochondrial CYPs form a unique branch in the phylogenic tree of animal CYPs [62]. To date, mitochondrial CYPs are only found in animals, but not in fungi and plants [63]. There is a minor group in the total P450 family members of animals compared with the microsomal CYPs. In *T. castaneum*, only nine out of 143 CYPs are found in mitochondria. In vertebrates, mitochondrial CYPs are generally specialized in the metabolism of steroid or vitamin D, in contrast with microsomal CYPs that show considerably extensive substrate specificities [1,62]. Whereas, insect mitochondrial CYPs show somewhat structural and functional diversity, which suggests that they have undergone several blooms [1,2]. There are at least two groups of mitochondrial CYPs in insects. One is CYP12 family including variable number of genes across different taxa that are rapidly evolving [1]. Three *CYP12A* genes were cloned from the house fly [41]. Among them, *CYP12A1* is constitutively overexpressed in diazinon resistant strain and metabolizes insecticides and other xenobiotics but not ecdysteroids. *D. melanogaster* has seven *CYP12* members in its CYPome (Figure 1B). The overexpression of *CYP12A4* in a natural population confers the lufenuron resistance [64]. *CYP12D1* was observed to be overexpressed in a DDT-resistant strain and induced by xenobiotics [42,65]. There are four *CYP12F* genes in *A. gambiae* CYPome (Figure 1B). It was reported that *CYP12F1* is constitutively overexpressed in both DDT-resistant strain (*ZAN/U*) and permethrin-resistant strain (*RSP*) [66]. The close association with xenobiotic resistance in the group of insect *CYP12* genes demonstrates the evolutionary differentiation between insects and vertebrates. The other group of insect mitochondrial CYPs show sequence conservation (Figure 1B) and include three Halloween genes that are the orthologs of the C22, C2, and C20 hydroxylases that function in the biosynthesis of ecdysteroids [3] as well as genes with unknown functions. These genes are considered to perform essential physiological functions during insect development and reproduction [1].

**Figure 6:**
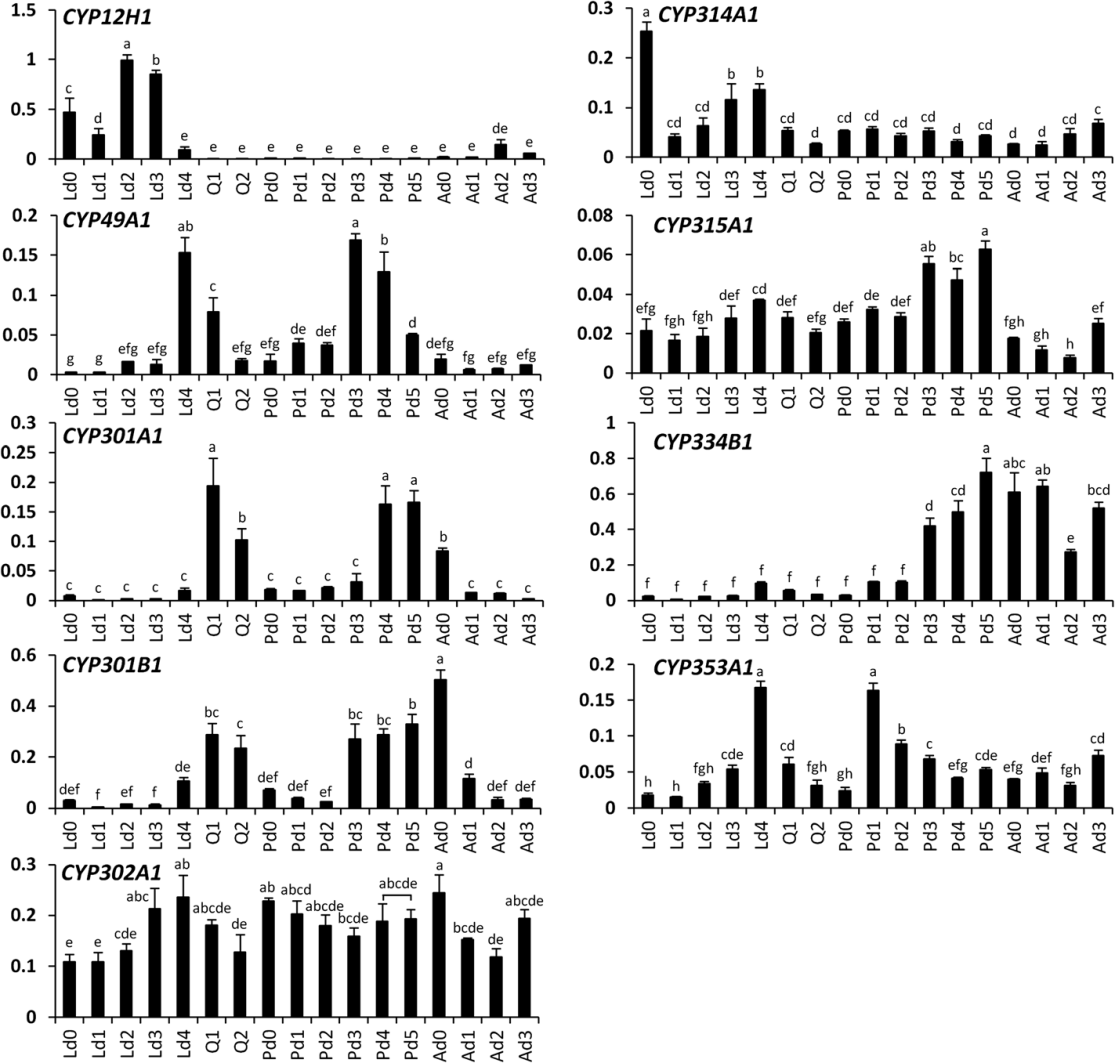
**Temporal expression patterns of *T. castaneum *mitochondrial CYPs.** The mRNA levels of nine *T. castaneum* mitochondrial CYPs in final instar larval stage (Ld0-Ld4), quiescent stage (Q1-Q2), pupal stage (Pd0-Pd5), and adult stage (Ad0-Ad3) were quantified and normalized using *rp49* as an internal control. There was no significant difference in the level of expression among samples designated with the same letter based on one-way ANOVA followed by Duncan multiple mean separation (SAS v9.4).

To predict the functions of *T. castaneum* mitochondrial CYPs, temporal expression of these genes was determined (Figure 6). Metamorphosis in holometabolous insects is regulated by cross-talk between ecdysteroids and juvenile hormones. Our previous studies showed that the ecdysteriod titers remain low throughout the final instar larval stage except for small increases at 60, 78 and 90 h after ecdysis into the final instar larval stage [67]. The ecdysteriod titers showed another peak during the quiescent stage. Afterward the levels remain low at the beginning of the pupal stage and increase again beginning at 42 h after ecdysis into the pupal stage and eventually reached the maximum levels by 66 h [67]. The mRNA levels of nine mitochondrial CYPs during the final instar larval stage (Ld0-Ld4), quiescent stage (Q1-Q2), pupal stage (Pd0-Pd5), and adult stage (Ad0-Ad3) were quantified and normalized using *rp49* mRNA levels as the most stable reference gene (Figure 6) [68]. *CYP12H1* is only expressed in the final instar larval stage which might point out that its function is restricted to this stage. The mRNA level of *shd* (*CYP314A1*) increased at the late period of the final instar larval stage at the time of the ecdysteriod increases. The expression of *sad* (*CYP315A1*) showed two peaks during the late periods of the final instar larval stage and pupal stage when the ecdysteriod titers reach the maximum levels (Figure 6). The mRNA levels of *CYP334B1* remained low until the late period of the pupal stage and afterward high levels were detected during the adult stage. The expression level of *CYP353A1* increased during the final instar larval stage and reached a peak in Ld4 and then Pd1 and subsequently decreased during the pupal and adult stages. *CYP302A1* gene is expressed ubiquitously.

**Figure 7:**
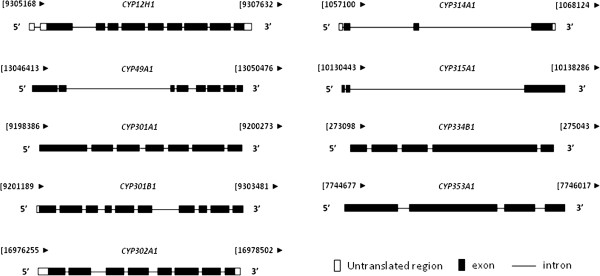
**Intron-exon arrangement of *T. castaneum *mitochondrial CYPs.** The white block represents the untranslated region, the black block shows the exon, and the black line strands the intron. Arrows indicate gene orientation: the left arrow is the reverse strand and the right arrow is the forward strand. The number beside the arrow shows the position of 5^′^ and 3^′^ ends on the chromosome.

The intron–exon organization of *T. castaneum* mitochondrial CYPs is also investigated which may help to understand the evolution of these genes as well as the origin of introns and genes [69]. As shown in Figure 7, intron-exon organization of all nine *T. castaneum* mitochondrial CYPs is highly diverse. The number and length of introns vary extensively among these genes. For example, *CYP301B1* contains nine small introns, whereas *CYP315A1* has only two introns. Other members contain 3-8 introns. Three Halloween genes share well conserved intron-exon positions among the different groups of orthologous genes [3]. However, *T. castaneum CYP314A1* and *CYP315A1* have undergone significant intron losses when compared to their orthologous genes. The massive intron loss is considered as a result of an evolutionary selection for compaction of insect genomes [70].

## Conclusions

In summary, integrated studies including annotation, phylogenetic analysis, gene expression as well as molecular modeling and docking were conducted for *T. castaneum* CYPs. 143 CYPs were identified and classified into 4 clans, 26 families and 59 subfamilies suggesting the CYP number in *T. castaneum* is three fold higher than honeybee and body louse, substantially higher than *D. melanogaster* and *A. gambiae* but significantly lower than *A. aegypti* and *C. quinquefasciatus*. The relatively large CYP gene superfamily in *T. castaneum* may contribute to the remarkable ability of insecticide resistance in this beetle. Current studies provided insights into the evolution of *T. castaneum* CYP gene superfamily and developed a valuable resource for the functional genomics research which will help to understand the strategies employed by insects to cope with their environment and to exploit potential new insecticide targets for pest control.

## Methods

### Red flour beetle strains

Three red flour beetle strains were used in this study. QTC279, originally collected from wheat storage in Malu, Queensland, Australia in 1984, was selected with pyrethroids for 10 generations until it was homozygous for the major pyrethroid resistance factor (2). LBS is an insecticide-susceptible strain. GA strain was used in whole genome sequencing project. These three strains were obtained from Dr. R.W. Beeman (U.S. Grain Marketing Research Laboratory of USDA, KS). Beetles were reared in whole wheat flour with brewers’ yeast (10% by weight) and maintained in darkness at 32°C and 55±2% relative humidity.

### Phylogenetic tree construction

All CYP sequences in insects which have the full open reading frames (ORFs) were extracted from the National Center for Biotechnology Information (NCBI) (Bethesda, MD) (http://www.ncbi.nlm.nih.gov/). The insect CYP amino acid sequences were analyzed using ClustalW alignment through Molecular Evolutionary Genetic Analysis software version 5 (MEGA 5) (http://www.megasoftware.net/) [71]. To significantly improve the alignments, the pair-wise alignment was performed with the gap opening penalty at 10 and the gap extension penalty left at default 0.1. The multiple alignment was conducted with the gap opening penalty at 3 and the gap extension penalty at 1.8 [72]. The sites containing obviously missing data or alignment gaps were eliminated in a pair-wise manner. A p-distance < 0.8 when carrying out the compute overall mean distance suggested the alignment was acceptable [72]. Subsequently, the alignment result was converted to a MEGA file (.meg) and submitted to construct the phylogenetic tree with neighbor-joining algorithm. A total of 2,000 bootstrap replications were used to test of phylogeny. Ultimately, the condensed tree was created with cut-off value of 50%.

### RNA extraction and quantitative real-time PCR (qRT-PCR)

Total RNA was extracted from adult beetles using TRI reagent (Molecular Research Center Inc., Cincinnati, OH). The qRT-PCR was performed in Applied Biosystems StepOnePlus™ Real-Time PCR System (Life technologies™, http://Carlsbad, CA). Total RNA was isolated from 3 adult beetles or 3-30 tissues for each sample and the RNA was treated with DNase I (Ambion Inc., Austin, TX). cDNA was synthesized using iScript cDNA synthesis kit (Bio-Rad Laboratories, Hercules, CA). DNase I treated total RNA was used as a template. Each qRT-PCR reaction (10 *μ*l final volume) contained 5 *μ*l FastStart SYBR Green Master (Roche Diagnostics, Indianapolis, IN), 1.0 *μ*l of cDNA, 3.6 *μ*l ddH_2_O, and 0.4 *μ*l each of forward and reverse gene specific primers (stock 10 *μ*M). An initial incubation of 95°C for 3 min, followed by 40 cycles of 95°C for 10 s, 55°C for 60 s settings were used. A fluorescence reading determined the extension of amplification at the end of each cycle. The most stable reference gene, *rp49*, was used for the housekeeping gene [68]. Both the PCR efficiency and R2 (correlation coefficient) value were taken into consideration in estimating the relative quantities. Each experiment was repeated at least three times using independent biological samples.

### Gene fragment isolation

Total RNAs were isolated from beetles in QTC279, GA, and LBS and cDNA was synthesized using iScript cDNA synthesis kit as described as above. The PCR products for CYP6BQ8, CYP6BQ9, CYP6BQ10 and CYP6BQ11 were amplified using primer pairs that were designed based on the sequences in NCBI database. The PCR products were cloned into pGEM®-T Easy Vector (Promega) and sequenced. Cloning and sequence analyses of P450 gene fragments were repeated at least three times with different preparations of RNAs. Three clones from each replication were sequenced.

### Deltamethrin induction experiments

One to two weeks old resistant QTC279 beetles were exposed to filter paper surface treated with deltamethrin [26,27]. According to preliminary study, 115 mg/48 cm^2^ deltamethrin that resulted in 50-60% mortality for QTC279 beetles was chosen for the experiment. The surviving beetles were collected for RNA extraction after 0, 6, 12, 24 h exposure to deltamethrin. The experiments were repeated three times. The statistical significance of the gene expression was calculated using a one-way analysis of variance (ANOVA) for multiple sample comparisons (SAS v9.4 software). A value of *P* ≤ 0.05 was considered statistically significant.

### Homology modeling and ligand docking

Initial protein models were constructed by submitting the translated amino acid sequences to the I-TASSER server [73]. The I-TASSER output includes up to 5 predicted models as well as predicted ligand binding sites. In the case of the P450 proteins modeled for this study the top scoring ligand binding site predictions all included heme bound to the conserved iron binding cysteine. For further model refinement the top scoring model was submitted to the FG-MD server for fragment guided molecular dynamics structure refinement [74]. The coordinates for heme were manually transferred to the refined model PDB file and a covalent bond was created between the heme iron and the conserved cysteine residue for each P450 modeled in this study. Model quality was examined by Ramachandran plots generated with Procheck [75]. Ramachandran plots of the P450 models gave a range of 97.8% to 98.9% of residues within the generously allowed regions and 2.2% or less in disallowed regions. Molecular docking was performed with Autodock Vina v1.1.2. [76]. Ligand (deltamethrin, permethrin, DDT, and imidacloprid) structures were retrieved from the Zinc database [77]. Proteins and ligands were prepared for docking with Autodock Tools v1.5.4 [78] For all dockings a search space with a grid box of 20 x 20 x 20 Å, centered at the heme bound Fe for each P450.

## Abbreviations

(CYP): Cytochrome P450; (RNAi): RNA interference; (20E): 20-hydroxyecdysone; (JH): Juvenile hormone; (CPR): NADPH-cytochrome P450 reductase; (ORF): Open reading frame; (qRT-PCR): quantitative real-time PCR

## Competing interests

The authors declare that there are no competing interests.

## Authors’ contributions

FZ, TWM and SRP designed research. FZ, TWM and KS performed experiments. FZ and TWM analyzed data. FZ, TWM and SRP wrote the paper. All authors read and approved the final manuscript.

## Supplementary Material

Additional file 1 **List of P450s in *Tribolium castaneum*.** Click here for file

Additional file 2 **Identity matrix of genes in the *CYP6BQ *cluster illustrating percentage identities among 12 cluster genes.** Click here for file

Additional file 3 **Intron-exon constructions of *CYP6BQ *cluster genes.** Shaded bars and lines represent gene exons and introns to scale, respectively. Click here for file

Additional file 4 **Differential expressions of clustered genes between resistant QTC279 and susceptible LBS strains.** The expression levels were normalized by *rp49*, the endogenous control. All data was averaged by three replicates. The result was shown as the mean + SE. Click here for file

Additional file 5 **Induction of clustered genes in QTC279 strain following treatment of deltamethrin.** The expression of these genes was analyzed by qRT-PCR as described in the methods. Relative expression level was normalized by *rp49*. The result was shown as the mean ±SEM (n= 3). There was no significant difference in the level of expression among samples with the same alphabetic letter (i.e. a, b, c) (*P* < 0.05). Click here for file

Additional file 6 **Sequence alignment for CYP6BQ8, 9, 10, 11.** Within 4.5 Å of deltamethrin, the predicted CYP6BQ8, CYP6BQ9, CYP6BQ10 and CYP6BQ11 catalytic sites contact with residues which were labeled in red color. Click here for file

Additional file 7 CYP6BQ cluster genes binding mode energy and distance. Click here for file
