# Supplementary material for: Integrated analysis of cytochrome P450 gene superfamily in the red flour beetle, Tribolium castaneum
Source: BMC Genomics. 2013 Mar 14;14:174. doi: 10.1186/1471-2164-14-174 (PMC3682917; doi:10.1186/1471-2164-14-174)
Supplement: Additional file 1 — List of P450s in Tribolium castaneum. [file 1471-2164-14-174-S1.pdf]

Additional file 1: List of P450s in *Tribolium castaneum*

No.	Clan	Name	Accession No.	Symbol Synonyms	Map position	Length (amino acid)
1	2	CYP15A1	EFA01264	GLEAN_02551	LG3	492
2	2	CYP18A1	EFA10669	GLEAN_01947	LG9	527
3	2	CYP303A1	EFA01261	GLEAN_02542	LG3	493
4	2	CYP304E1	EEZ99196	GLEAN_00411	LG2	501
5	2	CYP305A1	EFA01265	GLEAN_02552	LG3	493
6	2	CYP306A1	EFA10665	GLEAN_01916	LG9	481
7	2	CYP307A1	EFA11558	GLEAN_04159	LG1=X	535
8	2	CYP307B1	EFA05673	GLEAN_15030	LG6	487
9	Mito	CYP12H1	EFA07581	GLEAN_16365	LG7	535
10	Mito	CYP49A1	EFA07521	GLEAN_09877	LG7	539
11	Mito	CYP301A1	EFA02906	GLEAN_08302	LG4	522
12	Mito	CYP301B1	EFA02807	GLEAN_07167	LG4	513
13	Mito	CYP302A1	EFA04695	GLEAN_14691	LG5	510
14	Mito	CYP314A1	EEZ97722	GLEAN_11231	LG10	488
15	Mito	CYP315A1	EFA04669	GLEAN_14274	LG5	465
16	Mito	CYP334B1	EFA02864	GLEAN_07770	LG4	582
17	Mito	CYP353A1	EFA01331	GLEAN_03480	LG3	465
18	3	CYP6BK1	EFA12637	GLEAN_10256	Unknown	488
19	3	CYP6BK2	EFA12636	GLEAN_10255	Unknown	512
20	3	CYP6BK3	EFA12635	GLEAN_10254	Unknown	506
21	3	CYP6BK4	EFA12634	GLEAN_10253	Unknown	507
22	3	CYP6BK5	EFA12633	GLEAN_10252	Unknown	259
23	3	CYP6BK6	EFA12632	GLEAN_10238	Unknown	493
24	3	CYP6BK7	EFA12631	GLEAN_10239	Unknown	490
25	3	CYP6BK8P*	---	GLEAN_10241	Unknown	---
26	3	CYP6BK9P*	---	GLEAN_10242	Unknown	---
27	3	CYP6BK10	EFA12630	GLEAN_10243	Unknown	497
28	3	CYP6BK11	EFA05683	GLEAN_15102	LG6	503
29	3	CYP6BK12	EFA12529	GLEAN_03741	Unknown	491
30	3	CYP6BK13	EFA05693	GLEAN_15160	LG6	496
31	3	CYP6BK14	EFA05731	GLEAN_15551	LG6	488
32	3	CYP6BK15P*	---	GLEAN_03742	Unknown	---
33	3	CYP6BK16P*	---	GLEAN_15550	Unknown	---
34	3	CYP6BL1	EFA05684	GLEAN_15103	LG6	471
35	3	CYP6BM1	EFA12639	GLEAN_10236	Unknown	491
36	3	CYP6BN1	EFA05730	GLEAN_15546	LG6	485
37	3	CYP6BP1	EFA12638	GLEAN_10237	Unknown	475
38	3	CYP6BP2P	---	---	Unknown	---
39	3	CYP6BQ1	EFA02816	GLEAN_07310	LG4	513
40	3	CYP6BQ2	EFA02817	GLEAN_07311	LG4	520
41	3	CYP6BQ3P*	---	GLEAN_07312	LG4	---
42	3	CYP6BQ4	EFA02818	GLEAN_07313	LG4	520
43	3	CYP6BQ5	EFA02819	GLEAN_07314	LG4	519
44	3	CYP6BQ6	EFA02820	GLEAN_07315	LG4	520
45	3	CYP6BQ7	EFA02821	GLEAN_07316	LG4	519
46	3	CYP6BQ8	EFA02822	GLEAN_07317	LG4	516
47	3	CYP6BQ9	ADH29767	GLEAN_07318	LG4	521
48	3	CYP6BQ10	EFA02823	GLEAN_07320	LG4	520
49	3	CYP6BQ11	EFA02824	GLEAN_07321	LG4	520
50	3	CYP6BQ12	EFA02825	GLEAN_07322	LG4	521
51	3	CYP6BQ13	EEZ99338	GLEAN_04818	LG2	518
52	3	CYP6BQ14P*	---	GLEAN_09951	Unknown	---

53	3	CYP6BR1	EFA12629	GLEAN_10251	Unknown	494
54	3	CYP6BR2	EFA12628	GLEAN_10244	Unknown	499
55	3	CYP6BR3	EFA12627	GLEAN_10245	Unknown	497
56	3	CYP6BS1	EEZ99243	GLEAN_00856	LG2	514
57	3	CYP6BT1	EFA05733	GLEAN_15553	LG6	442
58	3	CYP345A1	EFA12856	GLEAN_00152	Unknown	505
59	3	CYP345A2	EFA12857	GLEAN_00153	Unknown	505
60	3	CYP345B1	EFA12855	GLEAN_00151	Unknown	506
61	3	CYP345C1	EFA12854	GLEAN_00148	Unknown	497
62	3	CYP345D1	EFA05713	GLEAN_15320	LG6	494
63	3	CYP345D2	EFA12532	GLEAN_03745	Unknown	492
64	3	CYP346A1	EFA04674	GLEAN_14346	LG5	502
65	3	CYP346A2	EFA04605	GLEAN_13433	LG5	497
66	3	CYP346B1	EFA04676	GLEAN_14359	LG5	503
67	3	CYP346B2	EFA04677	GLEAN_14360	LG5	493
68	3	CYP346B3	EFA04678	GLEAN_14361	LG5	503
69	3	CYP347A1	EFA04564	GLEAN_10955	LG5	520
70	3	CYP347A2	EEZ97687	GLEAN_16235	LG10	1390
71	3	CYP347A3	EEZ97687	GLEAN_10955	LG10	---
72	3	CYP347A4	XP_001809620	GLEAN_16235	LG10	490
73	3	CYP348A1	EFA07573	GLEAN_15916	LG7	428
74	3	CYP9D1	EFA09143	GLEAN_05354	LG8	463
75	3	CYP9D2	XP_001815543	GLEAN_05353	LG8	516
76	3	CYP9D3	EFA07793	---	LG8	965
77	3	CYP9D4	EFA09142	GLEAN_05352	LG8	524
78	3	CYP9D5	EFA09141	GLEAN_05351	LG8	523
79	3	CYP9D6*	---	---	Unknown	---
80	3	CYP9D7	EFA07804	GLEAN_05365	LG8	811
81	3	CYP9D8	XP_973646	---	LG8	379
82	3	CYP9W1	EFA09151	GLEAN_05387	LG8	480
83	3	CYP9X1	EFA09150	GLEAN_05386	LG8	507
84	3	CYP9Y1	XP_972348	GLEAN_06763	LG8	505
85	3	CYP9Z1	EFA09239	GLEAN_06441	LG8	497
86	3	CYP9Z2	EFA09240	GLEAN_06442	LG8	531
87	3	CYP9Z3	EFA09241	GLEAN_06443	LG8	504
88	3	CYP9Z4	EFA09242	GLEAN_06444	LG8	530
89	3	CYP9Z5	EFA09243	GLEAN_06445	LG8	503
90	3	CYP9Z6	EFA09275	GLEAN_15218	LG8	526
91	3	CYP9Z7	EFA09278	GLEAN_15487	LG8	484
92	3	CYP9AA1	EFA09276	GLEAN_15219	LG8	497
93	3	CYP9AB1	EFA09148	GLEAN_05384	LG8	530
94	3	CYP9AC1	EFA01242	GLEAN_00282	LG3	532
95	3	CYP9AD1	EEZ99187	GLEAN_00282	LG2	534
96	3	CYP9AF1	EFA09149	GLEAN_05385	LG8	477
97	4	CYP4G7	NP_001107860	GLEAN_07061	LG4	553
98	4	CYP4G14	NP_001107791	GLEAN_08058	LG4	560
99	4	CYP4Q1	EFA10754	GLEAN_12501	LG9	503
100	4	CYP4Q2	EFA10753	GLEAN_12500	LG9	504
101	4	CYP4Q3	EFA10752	GLEAN_12499	LG9	503
102	4	CYP4Q4	AAP94193	GLEAN_12503	LG9	491
103	4	CYP4Q5	EFA10757	GLEAN_12504	LG9	493
104	4	CYP4Q6	EFA10781	GLEAN_12662	LG9	496
105	4	CYP4Q7v1	EFA10755	GLEAN_12502	LG9	505
106	4	CYP4Q7v2*	AAP94192	---	LG9	505
107	4	CYP4Q8	XP_970987	GLEAN_12497	LG9	327
108	4	CYP4Q9P	EFA10751	GLEAN_12498	LG9	427

109	4	CYP4AA1	EFA01330	GLEAN_03478	LG3	482
110	4	CYP4BR1	EFA01322	GLEAN_03368	LG3	493
111	4	CYP4BR2P*	---	---	Unknown	---
112	4	CYP4BR3	EFA01323	GLEAN_03369	LG3	501
113	4	CYP4BM1	EFA07488	GLEAN_09412	LG7	511
114	4	CYP4BN1	NP_001123993	GLEAN_14125	LG5	506
115	4	CYP4BN2	EFA04617	GLEAN_13681	LG5	492
116	4	CYP4BN3	EFA04616	GLEAN_13680	LG5	499
117	4	CYP4BN4	EFA04615	GLEAN_13679	LG5	499
118	4	CYP4BN5	EFA09279	GLEAN_15992	LG8	498
119	4	CYP4BN6	EFA04535	GLEAN_10423	LG5	503
120	4	CYP4BN7	EFA04629	GLEAN_13773	LG5	487
121	4	CYP4BN8	EFA04628	GLEAN_13772	LG5	481
122	4	CYP4BN9	NP_001164235	GLEAN_02722	LG3	504
123	4	CYP4BN10	EFA01272	GLEAN_02721	LG3	501
124	4	CYP4BN11	NP_001123994	GLEAN_14641	LG5	492
125	4	CYP349A1	EFA05711	GLEAN_15296	LG6	488
126	4	CYP349A2	EFA05168	GLEAN_15295	LG6	814
127	4	CYP349A3P*	---	GLEAN_06829	Unknown	---
128	4	CYP350A1	EFA00865	GLEAN_03768	LG3	772
129	4	CYP350B1*	---	---	Unknown	454
130	4	CYP350C1	EEZ97716	GLEAN_11180	LG10	495
131	4	CYP351A1	EFA05167	GLEAN_15293	LG6	921
132	4	CYP351A2	EFA05709	GLEAN_15292	LG6	491
133	4	CYP351A3	EFA05708	GLEAN_15291	LG6	488
134	4	CYP351A4	EFA05707	GLEAN_15290	LG6	495
135	4	CYP351A5	EFA05706	GLEAN_15289	LG6	492
136	4	CYP351A6	EFA05705	GLEAN_15288	LG6	501
137	4	CYP351A7	EFA05704	GLEAN_15287	LG6	502
138	4	CYP351A8	EFA05703	GLEAN_15286	LG6	495
139	4	CYP351B1	XP_973400	GLEAN_15293	Unknown	1274
140	4	CYP351C1	EFA05717	GLEAN_15343	LG6	467
141	4	CYP351D1	EFA02923	GLEAN_15287	LG4	499
142	4	CYP352A1	EEZ99364	GLEAN_04973	LG2	495
143	4	CYP352A2	EEZ99363	GLEAN_04972	LG2	163

*More information is available in the P450 homepage: http://drnelson.uthsc.edu/CytochromeP450.html
