# Supplementary material for: Integrated analysis of cytochrome P450 gene superfamily in the red flour beetle, Tribolium castaneum
Source: BMC Genomics. 2013 Mar 14;14:174. doi: 10.1186/1471-2164-14-174 (PMC3682917; doi:10.1186/1471-2164-14-174)
Supplement: Additional file 2 — Identity matrix of genes in the CYP6BQ cluster illustrating percentage identities among 12 cluster genes. [file 1471-2164-14-174-S2.pdf]

Additional file 2. Identity matrix of genes in the CYP6BQ cluster illustrating percentage identities among 12 cluster genes. Values on the top right of the diagonal represent the identities of the full length of nucleotide sequence, while values on the bottom left are the amino acid sequence identities. Percent identities greater than 90% are shown in bold. “x” symbolizes no translated comparisons available for *CYP6BQ3P*.

	**CYP6BQ1**	**CYP6BQ2**	**CYP6BQ3**	**CYP6BQ4**	**CYP6BQ5**	**CYP6BQ6**	**CYP6BQ7**	**CYP6BQ8**	**CYP6BQ9**	**CYP6BQ10**	**CYP6BQ11**	**CYP6BQ12**
**CYP6BQ1**	-----	61	65	60	65	62	63	62	62	62	64	61
**CYP6BQ2**	52	-----	58	**94**	62	57	61	61	60	61	61	62
**CYP6BQ3**	x	x	-----	60	**95**	67	67	66	64	66	66	68
**CYP6BQ4**	52	**95**	x	-----	62	56	62	60	60	61	61	62
**CYP6BQ5**	61	56	x	56	-----	67	65	65	63	66	66	63
**CYP6BQ6**	59	56	x	57	67	-----	72	65	66	68	69	65
**CYP6BQ7**	57	57	x	57	64	75	-----	68	64	66	67	64
**CYP6BQ8**	58	52	x	53	62	63	63	-----	75	76	77	63
**CYP6BQ9**	58	53	x	55	61	64	61	70	-----	82	82	64
**CYP6BQ10**	59	56	x	56	65	64	64	73	76	-----	88	66
**CYP6BQ11**	60	55	x	55	66	66	65	75	79	87	-----	65
**CYP6BQ12**	55	53	x	54	60	65	63	61	60	62	63	-----
