# Supplementary figures and images for: Integrated analysis of cytochrome P450 gene superfamily in the red flour beetle, Tribolium castaneum
Source: BMC Genomics. 2013 Mar 14;14:174. doi: 10.1186/1471-2164-14-174 (PMC3682917; doi:10.1186/1471-2164-14-174)

## Additional file 3

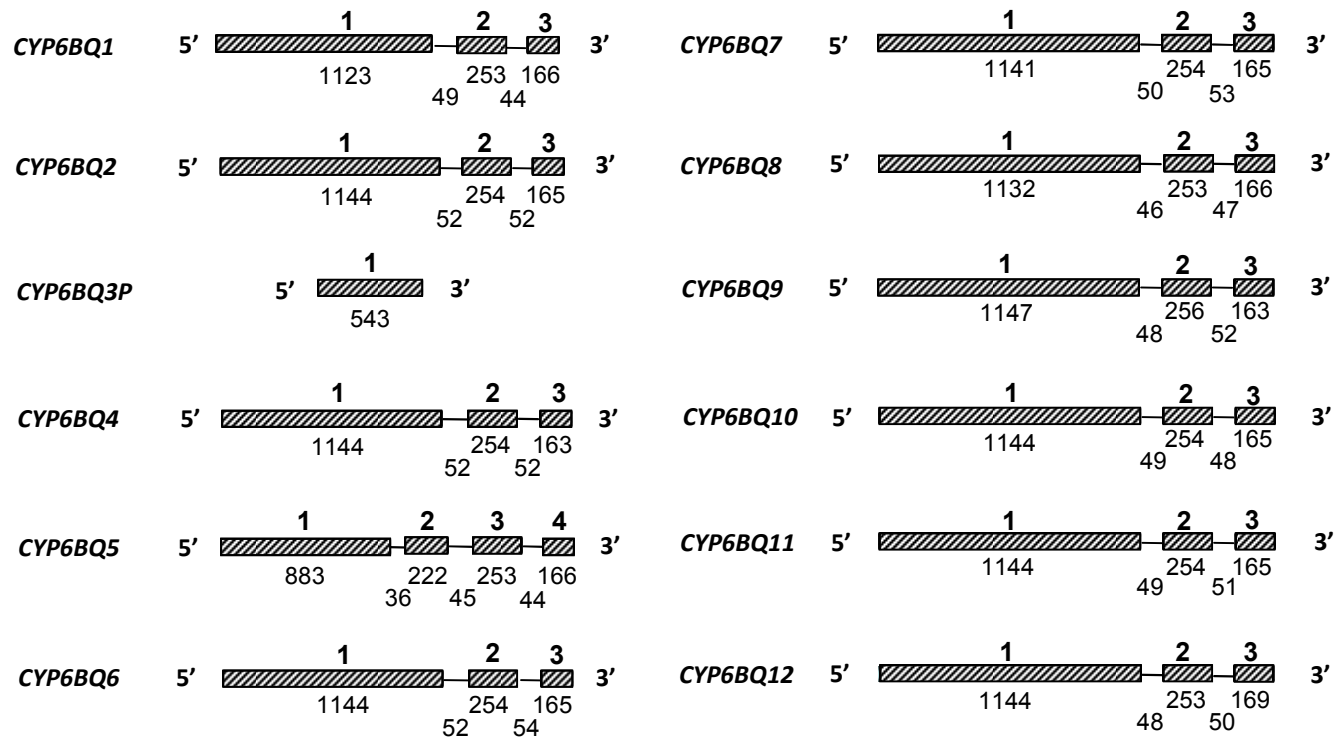

exon scale: 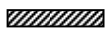 500bp    intron scale: 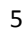 50bp

Supplement: Additional file 3 — Intron-exon constructions of CYP6BQ cluster genes. Shaded bars and lines represent gene exons and introns to scale, respectively. [file 1471-2164-14-174-S3.pdf]

## Additional file 4

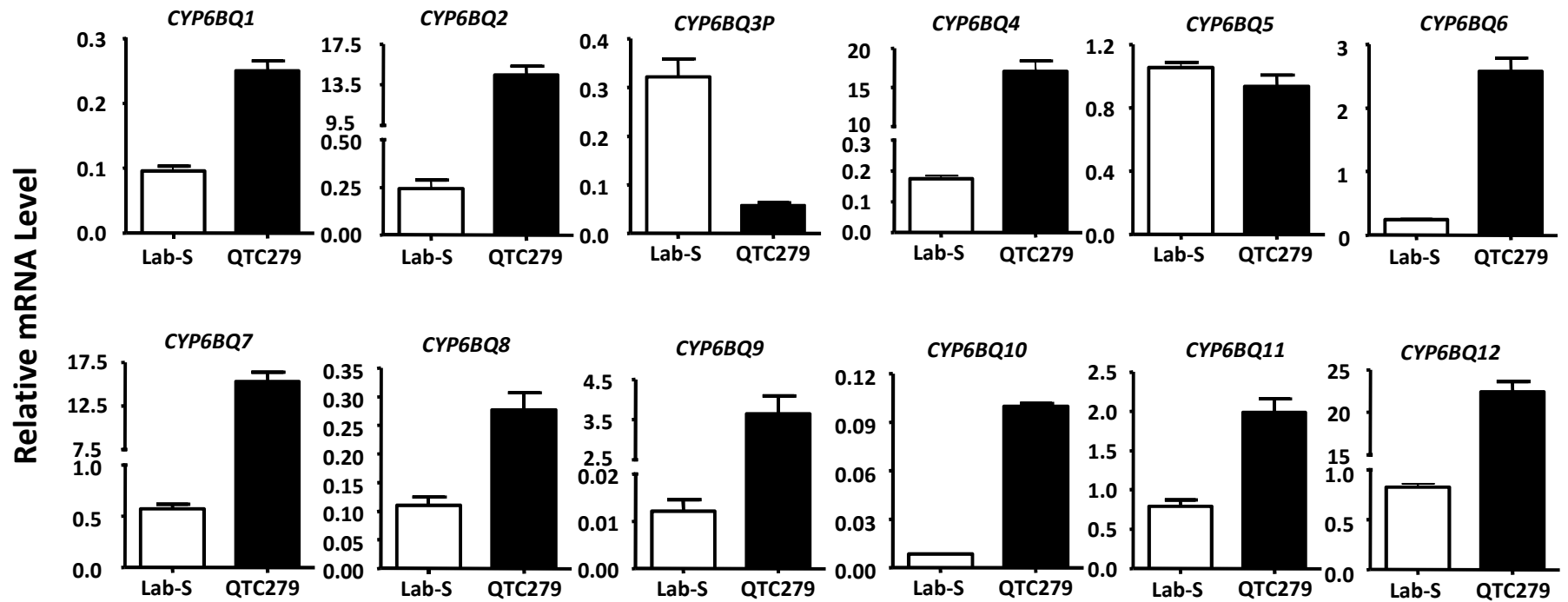

Supplement: Additional file 4 — Differential expressions of clustered genes between resistant QTC279 and susceptible LBS strains. The expression levels were normalized by rp49, the endogenous control. All data was averaged by three replicates. The result was shown as the mean + SE. [file 1471-2164-14-174-S4.pdf]

## Additional file 5

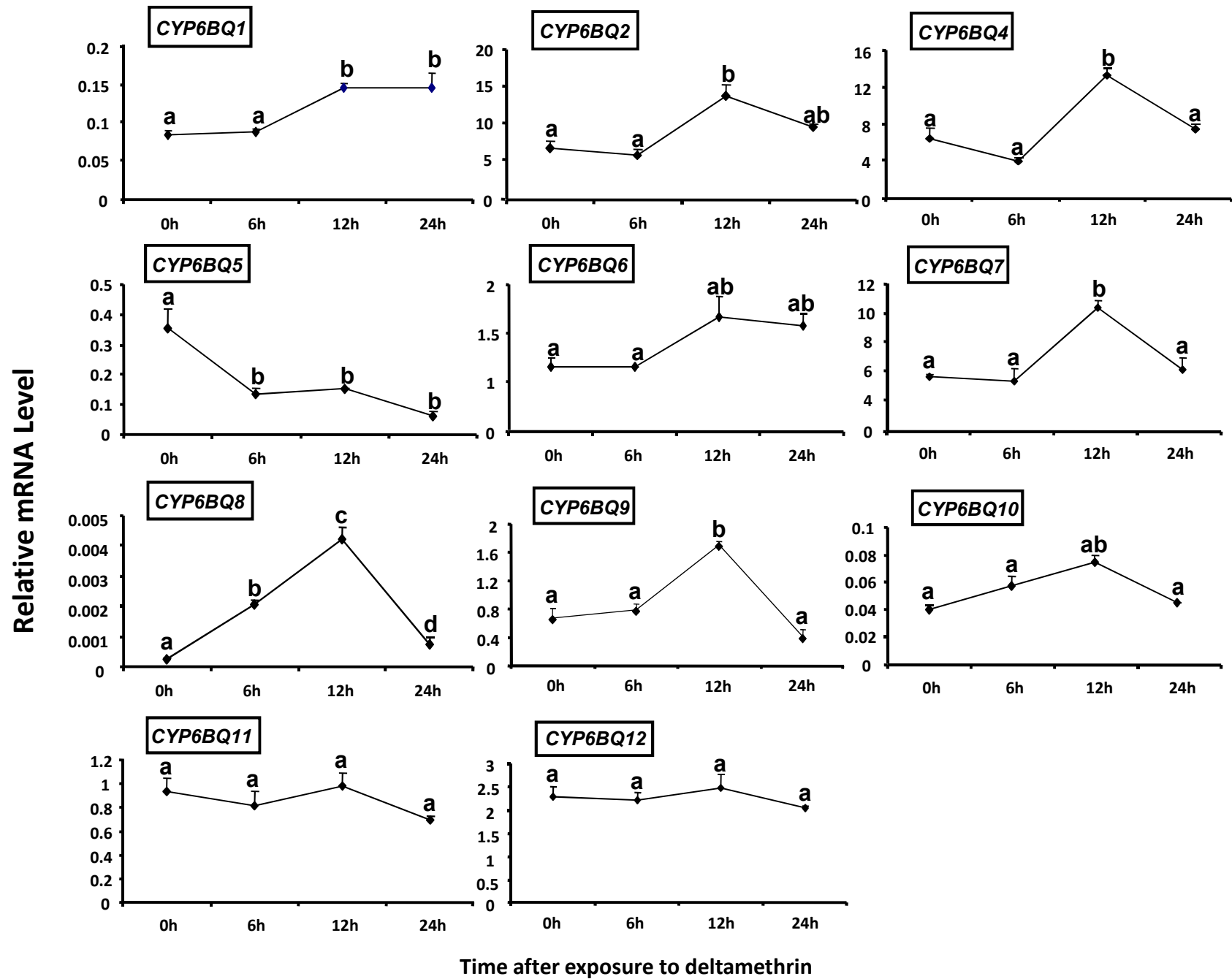

Supplement: Additional file 5 — Induction of clustered genes in QTC279 strain following treatment of deltamethrin. The expression of these genes was analyzed by qRT-PCR as described in the methods. Relative expression level was normalized by rp49. The result was shown as the mean ±SEM (n= 3). There was no significant difference in the level of expression among samples with the same alphabetic letter (i.e. a, b, c) (P < 0.05). [file 1471-2164-14-174-S5.pdf]
