# Supplementary material for: Integrated analysis of cytochrome P450 gene superfamily in the red flour beetle, Tribolium castaneum
Source: BMC Genomics. 2013 Mar 14;14:174. doi: 10.1186/1471-2164-14-174 (PMC3682917; doi:10.1186/1471-2164-14-174)
Supplement: Additional file 6 — Sequence alignment for CYP6BQ8, 9, 10, 11. Within 4.5 Å of deltamethrin, the predicted CYP6BQ8, CYP6BQ9, CYP6BQ10 and CYP6BQ11 catalytic sites contact with residues which were labeled in red color. [file 1471-2164-14-174-S6.pdf]

```
                          αA″   αA″      αA″       αA″      αA″      αA″
                        ~~~~~ ~~~~  ~~~~~~~~~~ ~~~~~~~~~~  ~        ~
CYP6BQ8-GA    1   ......MLLN NFTLN..... ......I... LAV..F.... .......... 14
CYP6BQ8-LBS   1   .......... .......... .......... .......... .......... 1
CYP6BQ8-PYR   1   .......... .......... .......... .......... .......... 1
CYP6BQ9-GA    1   .......... .......... .......... .......... .......... 1
CYP6BQ9-LBS   1   .......... .......... .......... .......... MTLIT.N.NV 8
CYP6BQ9-PYR   1   .......... .......... .......... .......... M......... 1
CYP6BQ10-GA   1   M....T.... .......... LITNNIT..L ...NLV..A. .......... 14
CYP6BQ10-LBS  1   .......... .......... .......... .......... .....MTLIT 5
CYP6BQ10-PYR  1   .......... .......... .......... .......... .......... 1
CYP6BQ11-GA   1   .MSLIT.... .....DNLTF ......DLIA ..I...LATI L......... 20
CYP6BQ11-LBS  1   .......... .......... .......... .......... .......... 1
CYP6BQ11-PYR  1   .......... .......... .......... .......... .......... 1

                   αA″  αA″       αA″     αA″      αA″   αA″  αA″     αA″
                  ~~~~~ ~~    ~~~~~~~~~~ ~~      ~~~ ~~~~~ ~~   ~~~~~~~~~~
CYP6BQ8-GA    14  .......... .......... .......... .......... .......... 14
CYP6BQ8-LBS   1   .......... .......... .......... .......... .......... 1
CYP6BQ8-PYR   1   .......... .......... .......... .......... .......... 1
CYP6BQ9-GA    1   .......... .......... .......... .......... ........M. 1
CYP6BQ9-LBS   9   TFDLIAIL.. .......... .......... .......A.. .......... 17
CYP6BQ9-PYR   1   .......... .......... .......... .......... .......... 1
CYP6BQ10-GA   14  ......I... .......... .......... .......... .......... 15
CYP6BQ10-LBS  5   ...N...... .......... .......... ........NI TLDFVAIIAT 18
CYP6BQ10-PYR  1   .......... .......... ..MTLITNNI TLDLVAII.. .......... 16
CYP6BQ11-GA   20  .......... .......... .......... .......... .......... 20
CYP6BQ11-LBS  1   ........MS LITDNLTFDL VA........ .......I.. .......... 15
CYP6BQ11-PYR  1   .......... .......... .......... .......... .........M 1

                  αA″         αA′      αA′  αA′     αA′     αA′        αA′
                  ~            ~~      ~~~~ ~~~~     ~~ ~~~~~~~~~~ ~~~~~~~~~
CYP6BQ8-GA    14  .......... .......... .......... .......... .......... 14
CYP6BQ8-LBS   1   .......... .......... ....MLLNNF TLNILA.VFV TI........ 17
CYP6BQ8-PYR   1   .......... ..MLLNNFTL NILA...... ..V..FITVL .V........ 19
CYP6BQ9-GA    1   ..T....... .......... .......... .......... .........L 3
CYP6BQ9-LBS   17  .......... .......... .......... .......... .......... 17
CYP6BQ9-PYR   1   .......... .......... .......... .......... .......... 1
CYP6BQ10-GA   15  .......... .......... .......... .......... .......... 15
CYP6BQ10-LBS  19  FLV....... .......... .......... .......... .........G 22
CYP6BQ10-PYR  16  .......... .......... .......... .......... .......... 16
CYP6BQ11-GA   20  .......... .......... .......... .......... .......... 20
CYP6BQ11-LBS  15  .......... .......... .......... .......... .......... 15
CYP6BQ11-PYR  1   ...SLITDNL TFN....... .......... .......... ..LVAILATI 19

                   αA′    αA′    αA′     αA′  αA′        αA′     αA′    αA′
                  ~~~~~     ~ ~~~~~~~~~~ ~~~ ~~ ~~~ ~.~.~~~~~~ ~~~   ~~~~~
CYP6BQ8-GA    14  ....V..... ..TIL..V.. .......... ..G....... ......V... 21
CYP6BQ8-LBS   17  .LV....... .G........ .........V ..IVY..... .........F 25
CYP6BQ8-PYR   19  .......... .......... .......GVI ......V... .......... 23
CYP6BQ9-GA    4   I.TNN..... LT..L...D. .......... ..L....... ......VAII 16
CYP6BQ9-LBS   17  .......... ........T. .......... .......LLV C.V....... 23
CYP6BQ9-PYR   2   T..L.ITNNL T....FDLI. A......... ..I.L....A ........TL 19
CYP6BQ10-GA   15  ....I..... A......T.. ....FLVG.. ..I..IV... .....Y.... 26
CYP6BQ10-LBS  22  .......... .......... .........I ..I....... ......I... 25
CYP6BQ10-PYR  16  .......... .........V T......... .......FLV GIIVYF.... 27
CYP6BQ11-GA   20  .......... ....V..G.. .......... ..I....... .....I..VY 26
CYP6BQ11-LBS  15  .......... .......... LATIL..... .VG....... .....II.VY 26
CYP6BQ11-PYR  20  L.V....... .......... .........G I.I....... .......... 24

                    αA′          αA′      αA′    αA′               αA
                   ~~~~~~~      ~~~~~~~ ~~~~~~~~~~ ~               ~~...~
CYP6BQ8-GA    21  .......... ......IVY. FKWHLSYWDR LGVPSLSPVL FFGDTK...D 51
CYP6BQ8-LBS   25  .......... .......KW. ...HLSYWDR LGVPSLSPVL FFGDTK...D 51
CYP6BQ8-PYR   23  .......... ...YF..KW. ...HLSYWDR LGVPSLSPVL FFGDTK...N 51
CYP6BQ9-GA    16  .ATF.LVGII VYF.K...W. ...QLSYWDR LGVPSLNPTL VFGDFK...N 53
CYP6BQ9-LBS   24  IA.Y...... .....L.KW. ...HLTYWNR LGVPSLNPVL FFGDAT...D 53
CYP6BQ9-PYR   19  .L.VCV...I AYL.K..... ..WHLTYWNR LGVPSLNPAL FFGDAT...D 53
CYP6BQ10-GA   26  .......... .......F.. .KWQLSYWDR LGVPSLNPTL VFGDFK...N 53
CYP6BQ10-LBS  25  .......... .........F FKWQLSYWDR LGVPSLNPAL IFGDFK...N 53
CYP6BQ10-PYR  27  .......... ........K. .W.QLSYWDR LGVPSLNPML VFGDFKNYIF 56
CYP6BQ11-GA   26  .......... .......... FKWQLSYWDR LGVPSLNPVL FFGDIK...D 53
CYP6BQ11-LBS  26  .......... .......... FKWQLSYWDR LGVPSLNPVL FFGDIK...D 53
CYP6BQ11-PYR  24  .......... .......VYF KW.QLSYWDR LGVPSLNPVL FFGDIK...D 53
```

```
                      αA          αA          αA              αA            β1-1
                     ~~~~        ~~         ~.~       ~ ~~~~~~~~~~~ ~~       →→
CYP6BQ8-GA     51  ....LILS..  ...K.C...T  I.G......E  QFRVFYNKF.  K.SKG.FKHG  78
CYP6BQ8-LBS    51  ....L.....  .I...LSKCT  ..I.GEQFRV  FYNKFKSKGF  .......KHG  78
CYP6BQ8-PYR    51  ....L.....  .I...L...S  KCTIGEQFRV  FYNKFKSKGY  .......KHG  78
CYP6BQ9-GA     53  ....Y.....  .IFAKC...S  L.G......E  QFKELYDQFK  SK...GYKHG  80
CYP6BQ9-LBS    53  ....Y.....  .L...FGKCG  V.G......E  QFAKFYGKFK  SK.G..YKHG  80
CYP6BQ9-PYR    53  ....YLFG..  ...K.C...G  V.G......E  QFAKFYGKFK  SK.G.Y.KHG  80
CYP6BQ10-GA    53  ....Y...IF  AK...C...S  L.G......E  QFKELYDQFK  ..SKG.YKHG  80
CYP6BQ10-LBS   53  .YIFA.....  .K...C...S  L.G......E  QFKELYDQFK  SK...GYKHG  80
CYP6BQ10-PYR   56  ....A.....  .K...C...S  L.G......E  QFKELYDQFK  SK...GYKHG  80
CYP6BQ11-GA    53  ...FI...LA  .K...C...T  L.G......E  QFKEFYGKFK  ..SKG.YKHG  80
CYP6BQ11-LBS   53  ....FILA..  ...K.C...T  L.G......E  QFKEFYGKFK  SK...GYKHG  80
CYP6BQ11-PYR   54  FI.LA.....  .K...C...T  L.G......E  QFKEFYGKFK  SK...GYKHG  80

                   β1-1   β1-2          αB      αB                    αB'      αB'
                   →→→→   →→→→    →→  ~~~~~~ ~~~~ ~~~~~               ~ ~   ~~~~~~
CYP6BQ8-GA     79  GIFFGPIPFY  IAIDPEIIKH  ILQKDFQHFM  NHG.YYI.N.  E.EDDPLTGH  124
CYP6BQ8-LBS    79  GIFFGPVPFY  IAIDPEIIKH  ILQKDFQHFM  NHG.YYI.NE  E..DDPLTGH  124
CYP6BQ8-PYR    79  GIFFGPIPFY  IAIDPEIIKH  ILQKDFQHFM  NHG.YYI.NE  E..DDPLTGH  124
CYP6BQ9-GA     81  GIFVGPKPFY  IPVDPDLGKH  IMQKDFQHFM  NHGFYIN.E.  E..VDPLTGN  126
CYP6BQ9-LBS    81  GVFLGPKCYY  VPVDPDLVKH  IMQKDFQHFM  NHG.FYINE.  E..VDPLTGN  126
CYP6BQ9-PYR    81  GVFLGPKCYY  VPVDPDLVKH  IMQKDFQHFM  NHGFYIN.E.  E..VDPLTGN  126
CYP6BQ10-GA    81  GIFVGPKPFY  VPIDPEIVQH  IMQKDFHHFM  NHG.NYF.D.  E.NADPLSGH  126
CYP6BQ10-LBS   81  GIFVGPKPFY  IPIDPEIVQH  IMQKDF.HHF  MNH.GNY.FD  E.NADPLSGH  126
CYP6BQ10-PYR   81  GIFVGPKPFY  IPIDPEIVQH  IMQKDF.HHF  MNH.GNY.FD  E.NADPLSGH  126
CYP6BQ11-GA    81  GIFLGPKPFY  VPIDPELVKH  IMQKDFQHFV  NHG.NYF.D.  E.DADPLSGH  126
CYP6BQ11-LBS   81  GIFLGPKPFY  VPIDPELVKH  IMQKDFQHFV  NHGNYFD.E.  D..ADPLSGH  126
CYP6BQ11-PYR   81  GIFLGPKPFY  VPVDPELVKH  IMQKDF.QHF  VNH.GNY.F.  DEDADPLSGH  126

                   αB'    αC      αC                αC'         αC'          αC'
                   ~~    ~~~~~~  ~~~~~~~~~~~  ~~~~~~~~~~~  ~~~~~~~~~~~  ~~~~~~~~~~~
CYP6BQ8-GA    125  LLNLENVKWK  NMRAKLTPTF  TSGKMKIMFQ  TLADYTTGLK  KVMDDSALNH  174
CYP6BQ8-LBS   125  LLNLENVKWK  NMRAKLTPTS  TSGKMKIMFQ  TLADYTTGLK  KVMDDSALNH  174
CYP6BQ8-PYR   125  LLNLENVKWK  NMRAKLTPTF  TSGKMKIMFQ  TLADCTTGLK  KVMDDSALNH  174
CYP6BQ9-GA    127  LFSLEDAKWR  NMRVKLTPTF  TSGKMKMMFQ  TLADCTRGLK  EIMDNLALNH  176
CYP6BQ9-LBS   127  LFSLEDAKWR  NMRVKLTPTF  TSGKMKMMFQ  TLADCTRGLK  EIMDNLALNH  176
CYP6BQ9-PYR   127  LFSLEDAKWR  NMRVKLTPTF  TSGKMKMMFQ  TLADCTRGLK  EIMDNLALNH  176
CYP6BQ10-GA   127  LFNLEDSKWK  NMRVKLTPTF  TSGKMKMMFQ  TLADCTRGLD  EIMDNSALNH  176
CYP6BQ10-LBS  127  LFNLEDSKWK  NMRVKLTPTF  TSGKMKMMFQ  TLADCTRGLD  EIMDNSALNH  176
CYP6BQ10-PYR  127  LFNLEDSKWK  NMRVKLTPTF  TSGKMKMMFQ  TLADCTRGLD  EIMDNSAVNH  176
CYP6BQ11-GA   127  LFNLEDAKWK  NMRIKLTPTF  TSGKIKMMFQ  TLADCTRGLK  EIMDHSALNH  176
CYP6BQ11-LBS  127  LFNLEDAKWK  NMRIKLTPTF  TSGKIKMMFQ  TLADCTRGLK  EIMDHSALNH  176
CYP6BQ11-PYR  127  LFNLEDAKWK  NMRIKLTPTF  TSGKIKMMFQ  TLADCTRGLK  EIMDHSALNH  176

                        αD        αD                            αD'          αE
                      ~~~~~ ~~~~~~~~~~~~ ~~     ~~~      ~~~~~~ ~~           ~
CYP6BQ8-GA    175  TPVDIKDIFG  RFTTDIIGSV  AFGIECNSLE  NPDAEFRKYG  KKVFEIDFFG  224
CYP6BQ8-LBS   175  TPVDIKDIFG  RFTTDIIGSV  AFGIECNSLE  NPDAEFRKYG  KKVFEIDFFG  224
CYP6BQ8-PYR   175  TPVDIKDIFG  RFTTDIIGSV  AFGIECNSLE  NPDAEFRKYG  KKVFEIDFFG  224
CYP6BQ9-GA    177  APADIKEILG  RFTTDIIGSV  AFGIECNSLK  NPDAEFRKYG  RKIVETGFID  226
CYP6BQ9-LBS   177  APADIKEILG  RFTTDIIGSV  AFGIECNSLK  NPDAEFRKYG  RKIVETGFID  226
CYP6BQ9-PYR   177  APADIKEILG  RFTTDIIGSV  AFGIECNSLK  NPDAEFRKYG  RKIVESGFID  226
CYP6BQ10-GA   177  APADIKDILG  RFTTDIIGSV  AFGIECNSLK  NPDAEFRKYG  RRVFEVGIID  226
CYP6BQ10-LBS  177  APADIKDILG  RFTTDIIGSV  AFGIECNSLK  NPDAEFRKYG  RRVFEVGIID  226
CYP6BQ10-PYR  177  TPADIKDILG  RFTTDIIGSV  AFGIECNSLK  NPDAEFRKYG  RRVFEVGIID  226
CYP6BQ11-GA   177  APADIKDILG  RFTTDIIGSV  AFGIECNSLK  DPDAEFRKYG  RKVFEVGF.I  225
CYP6BQ11-LBS  177  APADIKDILG  RFTTDIIGSV  AFGIECNSLK  DPDAEFRKYG  RKVFEVGF.I  225
CYP6BQ11-PYR  177  APADIKDILG  RFTTDIIGSV  AFGIECNSLK  DPDAEFRKYG  RKVFEVGF.I  225

                       αE          αE'              αF        αF          αF
                   ~~~~~~~~~~  ~~~~~~~~~~        ~~~~~~ ~~~~~~~~~~~ ~~~~~~
CYP6BQ8-GA    224  .RIKTLCTFA  IPHPILRLFR  FKFYNSDVAT  FFMDAIRETV  NYREKNNIYR  273
CYP6BQ8-LBS   225  RIKTLCT.FA  IPHPILRLFR  FKFYNSDVAT  FFMDAIRETV  NYREKNNIYR  273
CYP6BQ8-PYR   225  RIKTLCT.FA  IPHPILRLFR  FKFYNSDVAT  FFMDAIRETV  NYREKNNIYR  273
CYP6BQ9-GA    226  .RIKVFLVLS  IPHALLRFWR  FKFTNTEVET  FFMGAIQDTV  NYREKNNVYR  275
CYP6BQ9-LBS   227  RIKVFLV.LS  IPHALLRFWR  FKFTNTEVET  FFMGAIQDTV  NYREKNNVYR  275
CYP6BQ9-PYR   227  RIKVFLV.LS  IPHALLRFWR  FKFTNTEVET  FFMGAIQDTV  NYREKNNVYR  275
CYP6BQ10-GA   227  TIKIICI.LS  LPDSVLRLLK  LKFTKSDVEN  FFMNAIRDTV  NYREKNNIYR  275
CYP6BQ10-LBS  227  TIKIICILS.  LPHSVLRLLK  FRFTKSDVEN  FFMNAIRDTV  NYRE.KNNIY  274
CYP6BQ10-PYR  227  RIKIICILA.  LPDSVLRLLK  LKFTKSDVEN  FFMNAIRDTV  NYRE.KNNIY  274
CYP6BQ11-GA   226  DRLKTICILS  LPHPVLRFFK  LKFTKSDVEK  FFMSAIRDTV  NYREKNNIYR  275
CYP6BQ11-LBS  226  DRLKTICILS  LPHPVLRFFK  LKFTKSDVEK  FFMSAIRDTV  NYREKNNIYR  275
CYP6BQ11-PYR  226  DRLKTICILS  LPHPVLRFFK  LKFTKSDVEK  FFMSAIRDTV  NYRE.KNNIY  274
```

```
                             αG                αH           αH         αH   αH
                       ~~~~~~~~~~~~~~     ~~~~~~~~ ~~~~~~~~~~~ ~~~~~ ~~~
CYP6BQ8-GA     273 .KDFMHLLLQ LKNR...GLV .....TD.D. .....EK... .......... 294
CYP6BQ8-LBS    273 .KDFMHLLLQ LKNRG..LV. .....T..D. .......... .DE.....KI 295
CYP6BQ8-PYR    273 .KDFMHLLLQ LKNRG..LVT ......D.D. EK....I... ..T.G.D.KD 300
CYP6BQ9-GA     275 .KDFMHLLLQ LKNRG..LV. .....AD... .....D.... ........QK 296
CYP6BQ9-LBS    275 .KDFMHLLLQ LKNRGLVAD. .....DQKI. .....T.... ..D....... 299
CYP6BQ9-PYR    275 .KDFMHLLLQ LKNR...GLV ..AD....DQ KITD...... ...DKGN..I 304
CYP6BQ10-GA    275 .KDFMHLLLQ LKNRG..SV. .....TD... .D.E...... ....K.V.T. 298
CYP6BQ10-LBS   275 RKDFMHLLLQ LKNRG...S. .....VTD.. .D.EKV...T DD.....KDN 303
CYP6BQ10-PYR   275 RKDFMHLLLQ LKNRG...S. .....VT... ..D..D.... .E.......K 296
CYP6BQ11-GA    275 .KDFMHLLLQ LKNR...GFV A...DD..G. .....KV... .........T 298
CYP6BQ11-LBS   275 .KDFMHLLLQ LKNR...GFV ADDGK.V.T. .....D.... ........EK 301
CYP6BQ11-PYR   275 RKDFMHLLLQ LKNR...GFV A...D..... .DG...KVT. .D........ 299

                      αH    αH      αH             αI         αI         αI
                    ~~~~~~~ ~~ ~~~~~~~.~~      ~~~~~~ ~~~~~~~~~~ ~~~~~~~~~~
CYP6BQ8-GA     295 ITG...D... ..KDIV...T EALTMNELAA QAFVFFLAGF ETSSTAMTWA 333
CYP6BQ8-LBS    295 ...TGDKD.. .I...V...T EALTMNELAA QAFVFFLAGF ETSSTAMTWA 333
CYP6BQ8-PYR    300 .......... ..I..V...T EALTMNELAA QAFVFFLAGF ETSSTAMTWA 333
CYP6BQ9-GA     297 I.T...D.DK G....NIKEN DLITINELAA QAFVFFLGGF ETSSTTVSWA 337
CYP6BQ9-LBS    299 ...D..K... ..GN.IK.EN DLITINELAA QAFVFFLGGF ETSSTTMSWA 337
CYP6BQ9-PYR    304 .......... .K...E...N DLITINELAA QAFVFFLGGF ETSSTTMSWA 337
CYP6BQ10-GA    298 ...D.DKD.. ..NV.K...E KALTLNELSA QAFVFFLAGF ETSSTTMTWA 336
CYP6BQ10-LBS   303 .......... ..V.K....E KALTLNELSA QAFVFFLAGF ETSSTTMTWA 336
CYP6BQ10-PYR   297 VTD......D KDN.VK...E KALTLNELSA QAFVFFLAGF ETSSTTMTWA 336
CYP6BQ11-GA    298 ..DE..K... .EN.VK...E KALTLNELSA QAFVFFLAGF ETSSTTMTWA 336
CYP6BQ11-LBS   301 .......... ..ENVK...E KALTLNELSA QAFVFFLAGF ETSSTTMTWA 336
CYP6BQ11-PYR   300 E........K ..ENVK...E KALTLNELSA QAFVFFLAGF ETSSTTMTWA 336

                      αI     αJ     αJ                        αK         αK
                    ~~~~~~ ~~~ ~~~~~~~~~~ ~              ~~~~~~~~~~ ~~~~~~~~~~
CYP6BQ8-GA     334 LYELAINPDV QQKLRAEIND VLRKHNNKLT YEAMMDMTFM EKVICETLRK 383
CYP6BQ8-LBS    334 LYELAINPDV QQKLRAEIND VLRKHNNKLT YEAMMDMTFM EKVICETLRK 383
CYP6BQ8-PYR    334 LYELAINPDV QQKLRAEIND VLRKHNNKLT YEAMMDMTFM EKVICETLRK 383
CYP6BQ9-GA     338 LYELATNQDI QEKLRKEIND VLSRHNNKLS YDAMMEMTYM DKVINETLRK 387
CYP6BQ9-LBS    338 LYELATNQDI QEKLRKEIND VLSRHNNKLS YDAMMEMTYM DKVINETLRK 387
CYP6BQ9-PYR    338 LYELATNQDI QEKLRKEISD VLSRHNNKLS YDAMMEMTYM DKVINETLRK 387
CYP6BQ10-GA    337 LYELATNQDV QEKLRNEINN VLSRHDNKLT YEAMMEMTYM EKVIHETLRK 386
CYP6BQ10-LBS   337 LYELATNQNV QEKLRNEINN VLSRHDNKLT YEAMMEMTYM EKVIHETLRK 386
CYP6BQ10-PYR   337 LYELATNQNV QEKLRNEIIN VLSRHDNKLT YEAMMEMTYM EKVIHDTLRK 386
CYP6BQ11-GA    337 LYELATNQDV QEKLRNEINN VLSRHDNKLT YEAMMEMTYM EKVIHETLRK 386
CYP6BQ11-LBS   337 LYELATNQDV QEKLRNEINN VLSRHDNKLT YEAMMEMTYM EKVIHETLRK 386
CYP6BQ11-PYR   337 LYELATNQDV QEKLRNEINN VLNRYDNKLT YEAMMEMTYM EKVIHETLRK 386

                           β1-4    β2-1       β2-2     β1-3     αK'      αK'
                        →→→→ →   →→→       →→→     →→→ →→  ~~~~~~   ~~~
CYP6BQ8-GA     384 YPPIPVLTRK CTKDYTIPNT SIQLQRGVSV SVPVLALHTD PEYYPNPEKF 433
CYP6BQ8-LBS    384 YPPIPVLTRK CTKDYTIPNT SIQLQRGVSV SVPVLALHTD PEYYPNPEKF 433
CYP6BQ8-PYR    384 YPPIPVLTRK CTKDYTIPNT SIQLQRGVSV SVPVLALHTD PEYYPNPEKF 433
CYP6BQ9-GA     388 YPPLPIIPRV CNKDYTIPNT STKLSRGTSV AIPVLAIHTD PEYYPNPEKF 437
CYP6BQ9-LBS    388 YPPLPIIPRV CNKDYTIPNT STKLSRGTSV AIPVLAIHTD PEYYPNPEKF 437
CYP6BQ9-PYR    388 YPPLPIIPRV CNKDYTIPNT STKLSRGTSV AIPVLAIHTD PEYYPNPEKF 437
CYP6BQ10-GA    387 YPPLPILTRK CNKDYTIPNT CIKLRRGTTV AIPVLGLHTD PEYYSKPEKF 436
CYP6BQ10-LBS   387 YPPLPILTRK CNKDYMIPNT SIKLCRGTTV AIPVLGLHTD PEYYSNPEKF 436
CYP6BQ10-PYR   387 YPPLPILTRK CNKDYTIPNT SIKLRRGTTV AIPVLGLHTD PEYYSNPEKF 436
CYP6BQ11-GA    387 YPPLPILTRK CNKDYTIPNT SIKLSPGTAV GIPVLALHTD PEYYSNPEKF 436
CYP6BQ11-LBS   387 YPPLPILTRK CNKDYTIPNT SIKLSRGTAV GIPVLALHTD PEYYSNPEKF 436
CYP6BQ11-PYR   387 YPPLPILTRK CNKDYTIPNT SIKLSRGTAV GIPVLALHTD PEYYSNPEKF 436

                      αK"   αK"       αK"      Heme  αL          αL        αL    β3-3
                    ~~~~ ~~~~ ~~   ~~~~      ~~~~~~~~~~ ~~~~~~~~~~ ~~~~~~~~→→
CYP6BQ8-GA     434 DPDRFSDENV KARPGFTWLP FGEGPRICIG LRFGLLQSKV GLTAVLKHYR 483
CYP6BQ8-LBS    434 DPDRFSDENV KARPGFTWLP FGEGPRICIG LRFGLLQSKV GLTAVLKHYR 483
CYP6BQ8-PYR    434 DPDRFSDENV KARPGFTWLP FGEGPRICIG LRFGLLQSKV GLTAVLKHYR 483
CYP6BQ9-GA     438 DPEHFSEENV KARPGFTWLP FGDGPRICIG MRFGMMQSKV GLATILKNYK 487
CYP6BQ9-LBS    438 DPEHFSEENI KSRPGFTWLP FGDGPRICIG MRFGMMQSKV GLATILKNYK 487
CYP6BQ9-PYR    438 NPEHFNEENI KSRPGFTWLP FGDGPRICIG MRFGMMQSKV GLATILKNYK 487
CYP6BQ10-GA    437 DPEHFSEENV KSRPGFTWLP FGDGPRICIG LRFGMLQSKV GLTAILKNYK 486
CYP6BQ10-LBS   437 DPEHFSEENV KTRPGFTWLP FGDGPRICIG LRFGMLQSKV GLTAILKNYK 486
CYP6BQ10-PYR   437 DPEHFSEENV KSRPGFTWLP FGDGPRICIG LRFGMLQSKV GLTAILKNYK 486
CYP6BQ11-GA    437 DPEHFSEENV KTRPGFTWLP FGDGPRVCIG LRFGMLQSKV GLTALLKNYK 486
CYP6BQ11-LBS   437 DPEHFSEENV KTRPGFTWLP FGDGPRVCIG LRFGMLQSKV GLTALLKNYK 486
CYP6BQ11-PYR   437 DPEHFSEENV KTRPGFTWLP FGDGPRVCIG LRFGMLQSKV GLTALLKNYK 486
```

```
                     β3-3                          β3-2
                     →→                           → →→→
CYP6BQ8-GA     484 IKLNHKTQLP VTLNPRSF.I TSAKGGVWLD VEKID 517
CYP6BQ8-LBS    484 IKLNHKTQLP VTLNPR.SFI TSAKGGVWLD VEKID 517
CYP6BQ8-PYR    484 IKLNHKTQLP VTLNAR.SFI TSAKGGVWLD VEKID 517
CYP6BQ9-GA     488 IKLNNKTEFP IKVDPKNF.I TTAKGGVWLD VEKLD 521
CYP6BQ9-LBS    488 IKLNNKTEFP IKVDPK.NFI TTAKGGVWLD VEKLD 521
CYP6BQ9-PYR    488 IKLNNKTEFP IKVDPKNF.I TTAKGGVWLD VEKLD 521
CYP6BQ10-GA    487 VILSNKTKFP VTLDPK.SFI TTAKDGIWLD VKKLD 520
CYP6BQ10-LBS   487 VTLSNKTKFP VTLDPK.SFI TTAKDGIWLD VKKLD 520
CYP6BQ10-PYR   487 VTLSNKTKFP VTLDPK.SFI TTAKDGIWLD VKKLD 520
CYP6BQ11-GA    487 IKLSKKTELP IKLDPKSF.I TTAKGGIWLD VEKLD 520
CYP6BQ11-LBS   487 IKLSKKTELP IKLDPKSF.I TTAKGGIWLD VEKLD 520
CYP6BQ11-PYR   487 IKLSKKTELP IKLDPK.SFI TTAKGGIWLD VEKLD 520
```
